# Optimisation of Laser-Synthesized Heterostructured Multielement Nanoparticles for Solar Steam Generation and Water Purification

**DOI:** 10.1021/acsami.6c05452

**Published:** 2026-06-02

**Authors:** Runpeng Miao, Martina Roso, Linbo Jin, Marco Bortolussi, Ester Marotta, Vincenzo Amendola

**Affiliations:** † Department of Chemical Sciences, University of Padova, Via Marzolo 1, Padova 35131, Italy; ‡ Department of Industrial Engineering, University of Padova, Via Marzolo 9, Padova 35131, Italy

**Keywords:** laser ablation, DOE, nanoparticles, water evaporation, steam generation

## Abstract

The sustainable production of clean water is one major objective for both developing and industrialized regions. Nanoheterostructures offer synergistic optical and interfacial functionalities for water purification, yet their optimization against desired functions and sustainable synthesis protocols remains challenging. Here, we integrate design-of-experiments with laser ablation in liquids (LAL) to optimize multielement Fe–Mn–B nanoparticles (NPs) for interfacial solar steam generation and water purification. Multiobjective optimization identified a B-rich Fe_10_Mn_24_B_66_ LAL target composition as the Pareto-optimal region, and experimental validation further revealed that laser ablation in acetone produces a compositionally heterogeneous Fe/Mn/B@C nanoarchitecture with the highest photothermal output. When deposited onto cellulose support, the optimized NPs deliver an evaporation rate of 2.40 ± 0.05 kg·m^–2^·h^–1^ at 3.5 suns. Translation to electrospun nanofiber membranes enables >5× area scale-up without loss of evaporation rate, providing 0.56 ± 0.02 kg·m^–2^·h^–1^ under 1 sun, with a >6-fold enhancement relative to the membrane alone. The evaporator maintains desalination performance for 5–10 wt % NaCl brine and efficiently removes organic dyes and trace PFAS (PFOA), yielding low-conductivity, contaminant-free condensate. Finally, scenario-based life-cycle assessment identified electricity as the dominant hotspot at laboratory throughput and mapped a plausible pathway toward lower footprints as productivity and downstream manufacturing are improved.

## Graphical abstract

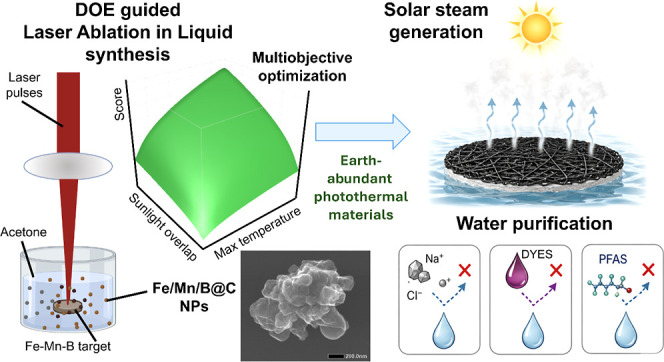

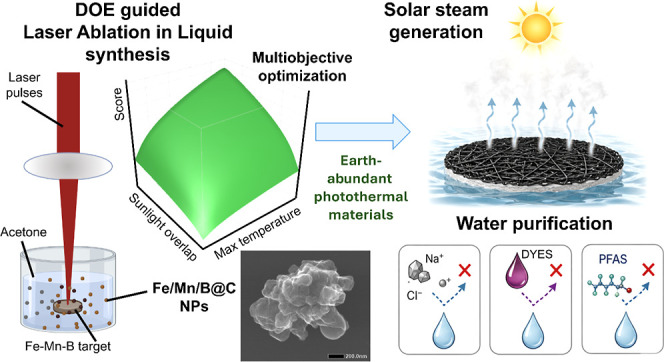

## 1 Introduction

Access to safe water is a defining challenge for both developing and industrialized regions, intensified by population growth, climate variability, and the persistence of hard-to-treat contaminants.^1^ Large-scale centralized treatment and distribution infrastructures are often economically or logistically prohibitive in remote settings, motivating compact, low-maintenance purification technologies that can operate off-grid.^2,3^ Solar-driven water distillation is particularly attractive because phase change intrinsically rejects nonvolatile solutes, enabling simultaneous desalination and removal of organic pollutants without reliance on high-pressure operation or consumable reagents.^4–7^ Consequently, the development of cost-effective solar steam generators has become a timely route toward decentralized water purification and desalination.^1^

Among solar-enabled approaches, interfacial solar steam evaporation (ISSG) has emerged as a powerful strategy to localize photothermal (PT) heating at the air–water interface, thereby minimizing bulk thermal losses and enabling high evaporation flux under natural sunlight.^8,9^ Over the past decade, substantial progress has been achieved through broadband absorbers and rational architectures that integrate optical trapping, thermal insulation, and capillary water transport.^4,6,9–13^ In this context, multiphase nanoparticles (NPs) assembled through internal interfaces (nanoheterostructures) have become essential building blocks across emerging green technologies, because the juxtaposition of dissimilar phases can yield synergistic optical, electronic, and chemical functions that are inaccessible to single-component nanocrystals.^14–17^

So far, the empirical approaches based on individual scientific experience have been the most frequent ones in designing and optimizing heterostructures for broadband absorption. Indeed, the simultaneous regulation of composition and functional parameters in heterostructures remains challenging because of the large number of combinations and variables, especially when considering wet-chemistry synthesis involving multiple reagents and experimental conditions.^18,19^ These materials-level constraints translate directly into deployment-level barriers for solar evaporators. Many high-performing devices are expensive because they rely on noble metals,^20^ complex nanostructures,^21^ multistep patterning.^22^ In other cases, chemical synthesis routes introduce toxic reagents and generate substantial solvent waste, resulting in a lack of sustainability. As the field moves from proof-of-concept devices toward scalable water treatment, the sustainability and manufacturability of both PT materials and membranes become constraints, as well as the evaporation flux itself.^23–27^ Recent studies indicate that the field is evolving beyond absorber optimization alone, with increasing emphasis on robust evaporator architectures, multifunctional water purification, and material stability under realistic operation. Examples include hierarchically porous hydrogel evaporators, photothermal evaporation–catalysis hybrid systems, and recent stability-focused assessments of photothermal materials for solar-heating water evaporation.^28–31^ Therefore, a critical yet under-addressed question is how the synthesis pathway of PT nanomaterials influences the overall environmental footprint of solar evaporators. Life-cycle assessment (LCA) of nanomaterials and membrane fabrication has repeatedly identified electricity, stock reagents and solvent supply chains as major contributors, particularly when laboratory-scale processes are operated at low yield.^32–34^

These concerns motivate the consideration of laser ablation in liquids (LAL) for the synthesis of heterostructured multielement nanoparticles for solar steam generation and water purification. LAL is a versatile platform that can generate surfactant-free colloidal NPs with high purity and absence of chemical precursors.^35–37^ LAL enables the one-step, chemistry-minimized realization of metastable compositions, multielement systems, and interfacial nanoarchitectures through ultrafast quenching of plumes laser-ablated from a bulk target.^35–38^ Laser-enabled strategies for solar steam generation are not limited to colloidal nanoparticle synthesis. Recent work has shown that femtosecond laser structuring of black absorbers can directly generate hierarchical three-dimensional carbon architectures for efficient solar steam generation, representing a complementary direct-patterning route to photothermal evaporators. In contrast, LAL offers distinctive advantages in surfactant-free colloid production, compositional tunability, and integration of multielement nanoheterostructures.^39^ Yet, classical LAL configurations are affected by low productivity, raising doubts about their scalability unless the throughput is improved via high-repetition-rate operation, optimized beam delivery, or multibeam/continuous-wave architectures.^40,41^ Despite rapid advances in both ISSG devices and LAL nanomaterial synthesis, an integrated strategy that connects materials discovery by LAL with ISSG device performance and prospective life-cycle implications remains unknown.^34,40,42^

These considerations suggest that the coupling of LAL to a data-driven, multiobjective optimization framework that explicitly balances PT performance against productivity and energy use can achieve high-performing PT nanomaterials but also a credible pathway toward scalable and low-impact manufacturing. Importantly, such an approach should treat NP composition, laser parameters, and solvent environment as jointly tunable variables that govern (i) optical absorption within the solar spectrum, (ii) heat generation, and (iii) colloid yield coupled with downstream processability.

Here, we report a design-of-experiment (DOE) guided LAL platform for optimizing multielement Fe–Mn–B PT NPs and translating them into scalable cellulose-based evaporators for solar steam generation and water purification. The Fe–Mn–B target family was selected as a low-cost, earth-abundant boride system in which boron-rich compositions were expected to support broadband absorption and structural robustness, while Fe and Mn provide transition-metal components capable of generating heterogeneous metallic/oxide domains and interfacial complexity under LAL. On this basis, DOE was used to identify the most favorable target composition within the Fe–Mn–B family, while solvent-controlled interfacial chemistry was subsequently exploited to access the final Fe/Mn/B@C nanoarchitecture. When integrated onto cellulose filters and scalable electrospun nanofiber membranes, the optimized coatings deliver high evaporation flux under concentrated illumination while retaining area-normalized performance upon scale-up. The NP-cellulose membrane achieves efficient rejection of dyes, salt and persistent contaminants such as PFAS, highlighting broad-spectrum purification capability. Finally, we quantify how LAL productivity governs the climate footprint of the evaporator via a scenario-based LCA, mapping a plausible transition toward industrially relevant impacts. Collectively, this work positions LAL as a viable, chemistry-minimized route to scalable PT nanoheterostructures, as far as laser productivity and downstream manufacturing are improved, delineating pathways toward lower-impact devices for ISSG applications.

## 2 Results

### 2.1 General Approach

The approach to the optimization of the PT conversion ability and stability of heterostructured multielement NPs using LAL is summarized in Figure 1. It relies on the systematic exploration of the influence of target and solvent composition and laser pulse duration on three performance indicators relevant for the intended PT application: the reached temperature peak of NP under broadband illumination (temperature peak); NP yield per synthesis (concentration indicator); spectral overlap with the solar spectrum (convolution area). A total of 24 experiments were designed (see Table S1 in Supporting Information), varying target atomic composition (Fe_2_B, FeB_2_, Mn_2_B, MnB_2_) and solvent (water, water/ethanol 1:1, ethanol), producing 12 Fe–B and 12 Mn–B NP samples. The performance indicators of the 24 samples were subsequently characterized by UV–vis absorption spectroscopy and PT experiments. This resulted in the experimental data set comprising input factors (target and solvent compositions, pulse duration) and output response (peak temperature, concentration indicator, convolution area). Then, a DOE regression analysis was performed to model the relationship between synthesis conditions and PT performance. This analysis yielded a well-fitted mathematical function that captures the correlation between experimental parameters and performance indicators. Using the second-order (quadratic) response-surface model, an inverse prediction approach on the synthesis parameters was applied to simultaneously optimize all three indicators, aiming to achieve high-performance solar water evaporation using low-cost and earth-abundant NP composition. The Fe–Mn–B system was chosen as the initial target family because it combines earth-abundant transition metals with a boron-rich component expected to favor broadband photothermal response and structural stability, while still offering sufficient compositional freedom for DOE-guided optimization.

**Figure 1:**
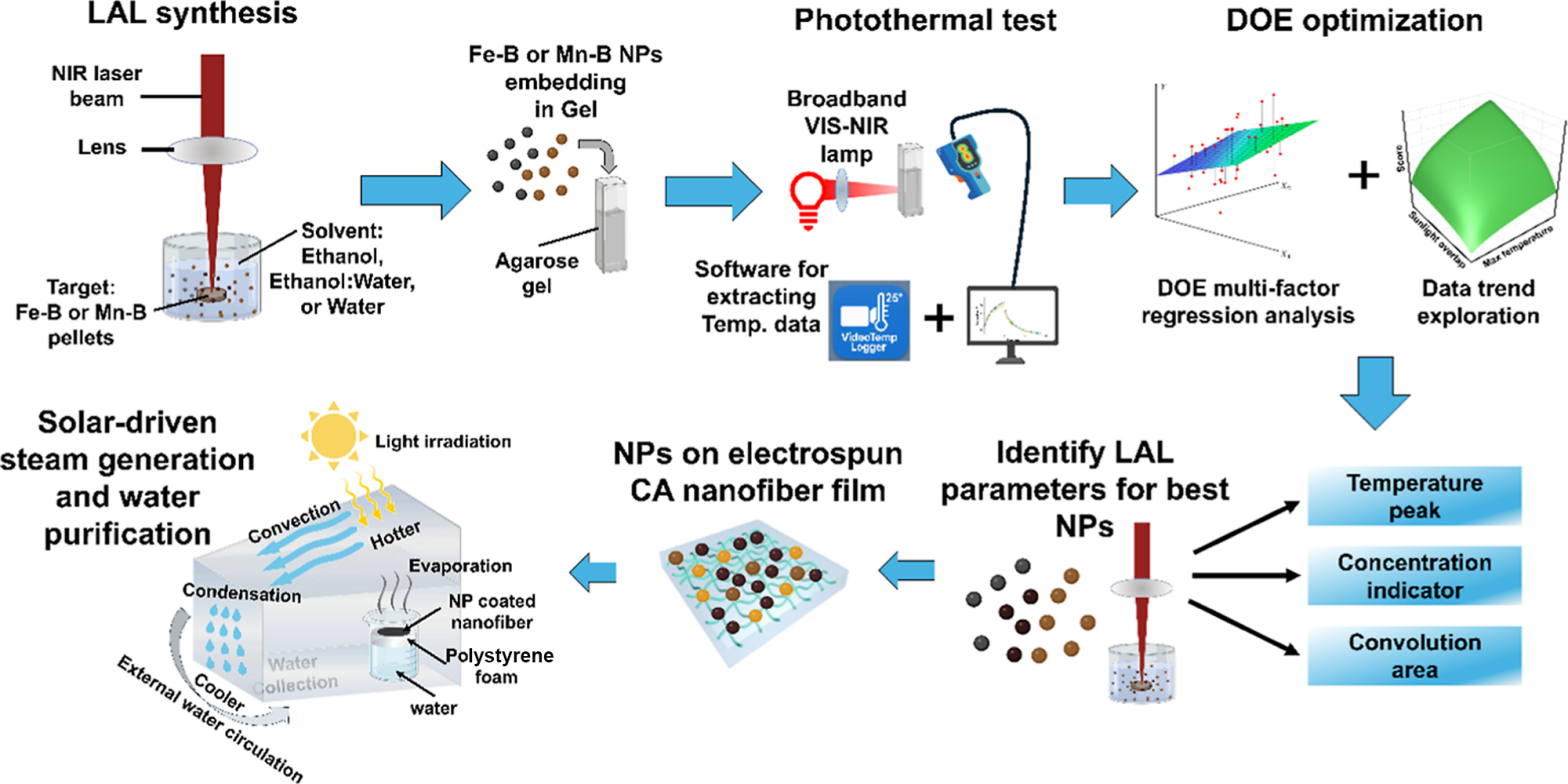
Schematic representation of the experimental and data-driven workflow for optimization of multielement NP synthesis with PT properties for ISSG applications.

### 2.2 NPs Optical Absorption versus LAL Synthesis Parameters

The 24 samples of Fe–B and Mn–B, synthesized according to the parameters reported in Table S1, were collected by centrifugation and resuspended in pure water for comparative UV–vis analysis (Figure 2A). Optical absorption of the Fe–B and Mn–B NPs is significantly affected by LAL synthesis parameters, as shown in Figure 2B–E. Changing the pulse duration from 20 to 200 ns generally results in the red-shifted absorption and spectral broadening, although the target composition and solvent environment also play a relevant role. For instance, Fe_2_B NPs synthesized under long-pulse conditions in ethanol (sample 4, 200 ns, see Table S1 for the complete list of LAL parameters) exhibit the broadest and most red-shifted absorbance within the Fe_2_B series (Figure 2B). Conversely, for Fe_2_B NPs synthesized in water with a 200 ns pulse duration (sample 6), broadband absorption was the lowest among the tested conditions (Figure 2B), likely due to the combined effects of target composition and the oxidative aqueous environment. Similarly, FeB_2_ and MnB_2_ samples prepared with 200 ns pulses in pure water (samples 6 in, respectively, Figure 2C,E) display broadened spectra with pronounced NIR background absorption in their respective groups. FeB_2_ synthesized with a 20 ns pulse in water (sample 3 in Figure 2C) and MnB_2_ synthesized with a 20 ns pulse in 1:1 ethanol-to-water mixture (sample 2 in Figure 2E), exhibit the lowest overall absorbance and a suppressed NIR optical density among, respectively, the FeB_2_ or MnB_2_ series, suggesting that short-pulse ablation in an oxidative medium is not optimal for the broadband optical properties. Interestingly, the Mn_2_B sample synthesized under short-pulse conditions in ethanol (sample 1 in Figure 2D) deviates from this trend, showing an unexpectedly broad absorption spectrum and large optical density in the NIR. This anomaly can be attributed to the intrinsic physicochemical properties of Mn combined with the less oxidizing conditions under shortest-pulse irradiation. Mn has a lower melting point (1246 °C) than Fe (1538 °C) and B (2076 °C), which was associated with more efficient ablation for some metals.^43,44^ The ethanol solvent, rich in organic radicals, effectively stabilizes the NPs by limiting oxidation and fostering aggregation, resulting in broadened optical absorption.

**Figure 2:**
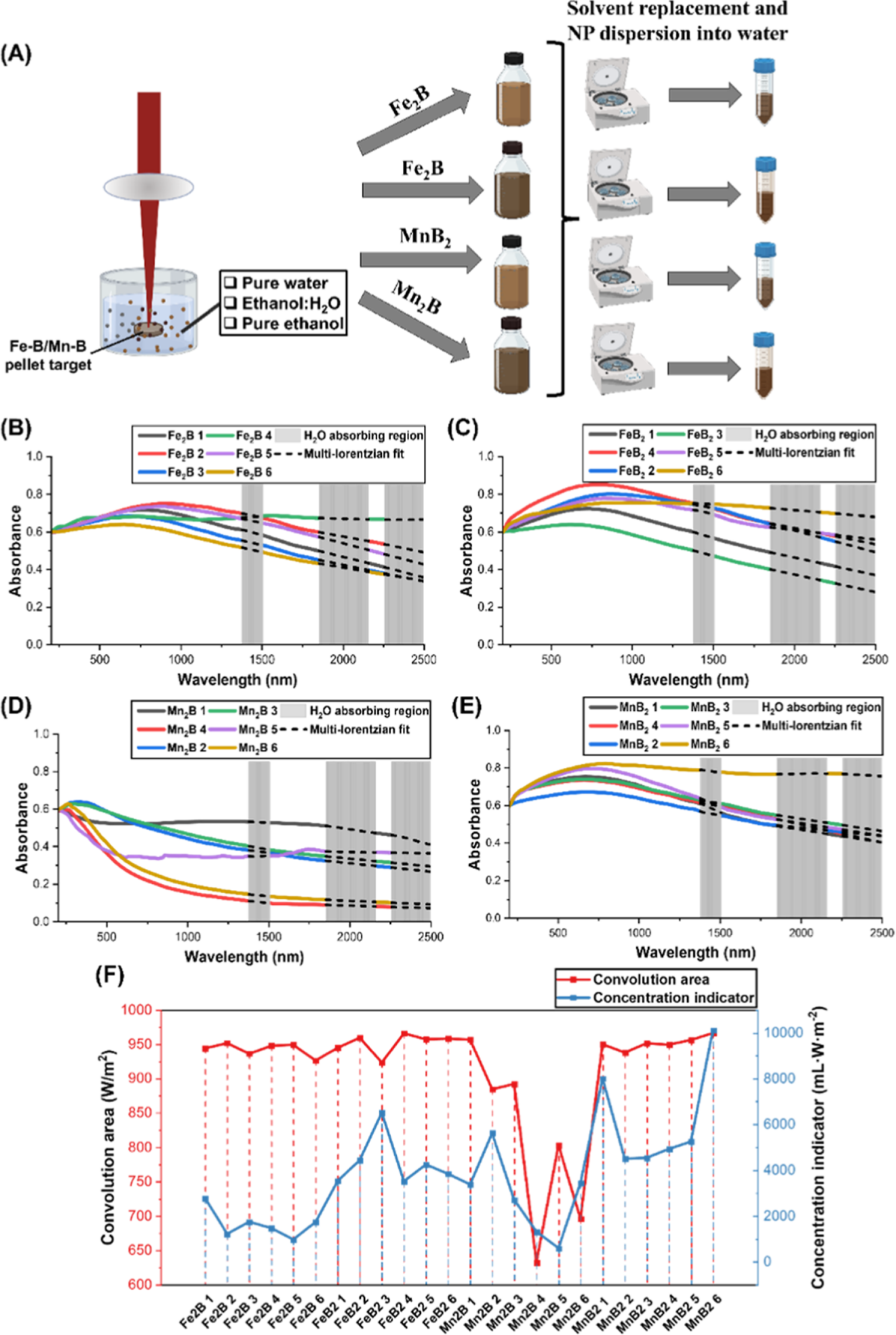
(A) Schematic of the LAL synthesis of Fe–B and Mn–B NPs. (B–E) Absorbance spectra for Fe_2_B (B), FeB_2_ (C), Mn_2_B (D) and MnB_2_ (E) NPs. The spectral range extends to 2500 nm to allow comparison with solar emission. All solutions were set with the same absorbance value of 0.6 at 200 nm for the sake of comparison and for the other experiments and metric calculations. (F) The convolution area and concentration indicator of Fe_2_B, FeB_2_, Mn_2_B and MnB_2_ NPs series.

### 2.3 Metrics Calculation and PT Characterization for Data Set Building

Then, the “convolution area” (W·m^–2^) of the UV–vis–NIR NP absorbance (after setting the absorbance at 200 nm to the standard value of 0.6 in a 2 mm quartz cuvette) with solar irradiance at AM 1.5 was considered as one of the three NP performance metrics toward ISSG applications. The second NP performance metric was the “concentration indicator”, obtained by multiplying NP solution volume (in mL) with the convolution area (in W·m^–2^) in the visible range (380–780 nm), reflecting the efficiency in LAL synthesis of NP with useful optical properties for solar light absorption. The choice of the visible range was motivated by the need to avoid the irrelevant contribution of absorption peaks in the UV (as in samples Mn_2_B 4 and 6 in Figure 2D), which do not overlap with solar emission, while also avoiding the NIR region, which overlaps with water absorption bands. The third and last metric consists of the reached “temperature peak” of all synthesized Fe–B and Mn–B NP samples under broadband visible-NIR irradiation. These three indicators were selected because, in our synthesis endeavor, they form the minimal yet sufficient set to constrain ISSG-relevant performance and manufacturability. The “convolution area” with the solar spectrum quantifies light-harvesting potential, the NP “concentration indicator” captures productivity that ultimately governs scalability and sustainability, and the “peak temperature” serves as an integrated proxy for net PT conversion under a standardized geometry. Together, they span the key trade-offs that determine whether a synthesis condition is attractive for scalable ISSG, while additional descriptors (e.g., size, aggregation, phase fraction) primarily act as mechanistic factors that manifest through one or more of these indicators.

The variation of NP performance metrics with the synthesis conditions is shown in Figures 2F and 3. The convolution of absorbance spectra with the solar irradiation (Figure 2F) is similar in magnitude among the Fe–B samples, indicating that both Fe_2_B and FeB_2_ series exhibit stable and broadband absorption profiles. Instead, the synthesis yield of broadband absorbing NPs is higher for FeB_2_ than Fe_2_B samples. A different behavior is observed for the MnB_2_, which displays markedly lower yields and narrower absorption spectra than the Mn_2_B samples. Furthermore, samples 1–3 of the Mn_2_B series (20 ns pulses in, respectively, pure ethanol, ethanol–water 1:1, and pure water) exhibited concentration indicator values of 3.37 × 10^3^ mL·W·m^–2^, 5.61 × 10^3^ mL·W·m^–2^, and 2.70 × 10^3^ mL·W·m^–2^, whereas their long-pulse counterparts (samples 4–6) yielded generally lower values of 1.31 × 10^3^ mL·W·m^–2^, 5.87 × 10^2^ mL·W·m^–2^, and 3.44 × 10^3^ mL·W·m^–2^, respectively. These results indicate that short-pulse ablation, particularly in ethanol–water mixtures, is beneficial for Mn–B NP productivity. The corresponding convolution areas, expressed in W·m^–2^, followed a similar trend, with short-pulse Mn_2_B samples (956.98, 884.25, and 892.00 W·m^–2^ for samples 1–3) exceeding their long-pulse analogues (632.02, 803.01, and 696.13 W·m^–2^ for samples 4–6). This trend could be due to the Mn higher tendency to oxidation during prolonged laser irradiation.^43,44^ Overall, the convolution area and concentration indicator suggest that higher contents of boron are associated with better metrics. However, the optical absorption spectra alone do not provide information on the PT performance of the synthesized NPs, because they do not discriminate between pure absorption and scattering. Hence, the PT properties of the different Fe–B and Mn–B NPs were scrutinized after embedding the colloids into an agarose gel matrix, providing the third performance metric. All colloidal suspensions were used after adjusting their absorbance to the same value at 200 nm, consistent with the calculation of the two previous performance metrics. As schematized in Figure 3A,B, the NP dispersions were mixed with a hot aqueous agarose solution and cast into cuvettes, where gelation immobilized the NPs within a three-dimensional polymer network. Embedding the NPs in agarose suppresses convective flows during irradiation and prevents sedimentation, which both improves PT energy retention in the probed volume and enhances measurement reproducibility, providing a reliable platform for controlled PT studies.^45^ The irradiation was executed using a broadband and continuous wave lamp at a light intensity of 2.51 ± 0.01 W/cm^2^ across the UV–vis–NIR (99% of emission from 370 to 2500 nm). The reached temperature after 20 min of light irradiation was registered as the third NP performance metric of interest for the ISSG application. Photographs of all gel-embedded samples, together with their PT heating–cooling curves, absorbance spectra before and after irradiation, and peak temperatures plot, are reported in Figures S1–S8 in Supporting Information. The heating–cooling profiles and pre/postirradiation absorbance spectra of the three best-performing samples are shown in Figure 3C–H. The PT heating curves for the Fe_2_B gel reveal a certain dependence on the synthesis parameters (Figure S1A–F). For NPs generated with a short pulse duration (20 ns), the highest temperature of 61.6 ± 0.3 °C was achieved with the sample synthesized in H_2_O/ethanol mixture (sample 2), followed by the pure ethanol (sample 1, 60.9 ± 0.5 °C) and pure H_2_O (sample 3, 59.5 ± 0.1 °C). A similar trend was observed for the series synthesized with a longer pulse duration (200 ns), where pure ethanol (sample 4) yielded the most efficient PT agent with the temperature peak of 62.9 ± 0.3 °C. Notably, the sample prepared in the H_2_O/ethanol mixture with a 200 ns pulse duration (sample 5) exhibited the lowest heating efficacy of the Fe_2_B series, with the temperature peak of 58.6 ± 0.4 °C. All six samples demonstrated good PT stability, as evidenced by the high degree of overlap between the absorbance spectra of first and second heating–cooling cycles (Figure S2A–F). The PT performance of the FeB_2_ series shows better PT trends (Figure S3A–F). Among the samples synthesized with a 20 ns pulse duration, the colloid prepared in the 1:1 H_2_O/ethanol mixture (sample 2) demonstrated improved performance, reaching the second-highest temperature peak of 64.0 ± 0.5 °C among all samples (Figure 3D). The sample from pure ethanol (sample 1) reached 61.8 ± 0.1 °C, while the one from pure H_2_O (sample 3) showed the lowest performance at 59.6 ± 0.2 °C. Conversely, for the samples synthesized with a 200 ns pulse duration, the trend was inverted; the NP colloid prepared in pure H_2_O (sample 6) achieved the highest temperature elevation (63.3 ± 0.6 °C). The consistency between the two measurement cycles for six samples confirms their good stability as PT agents (Figure S4A–F).

**Figure 3:**
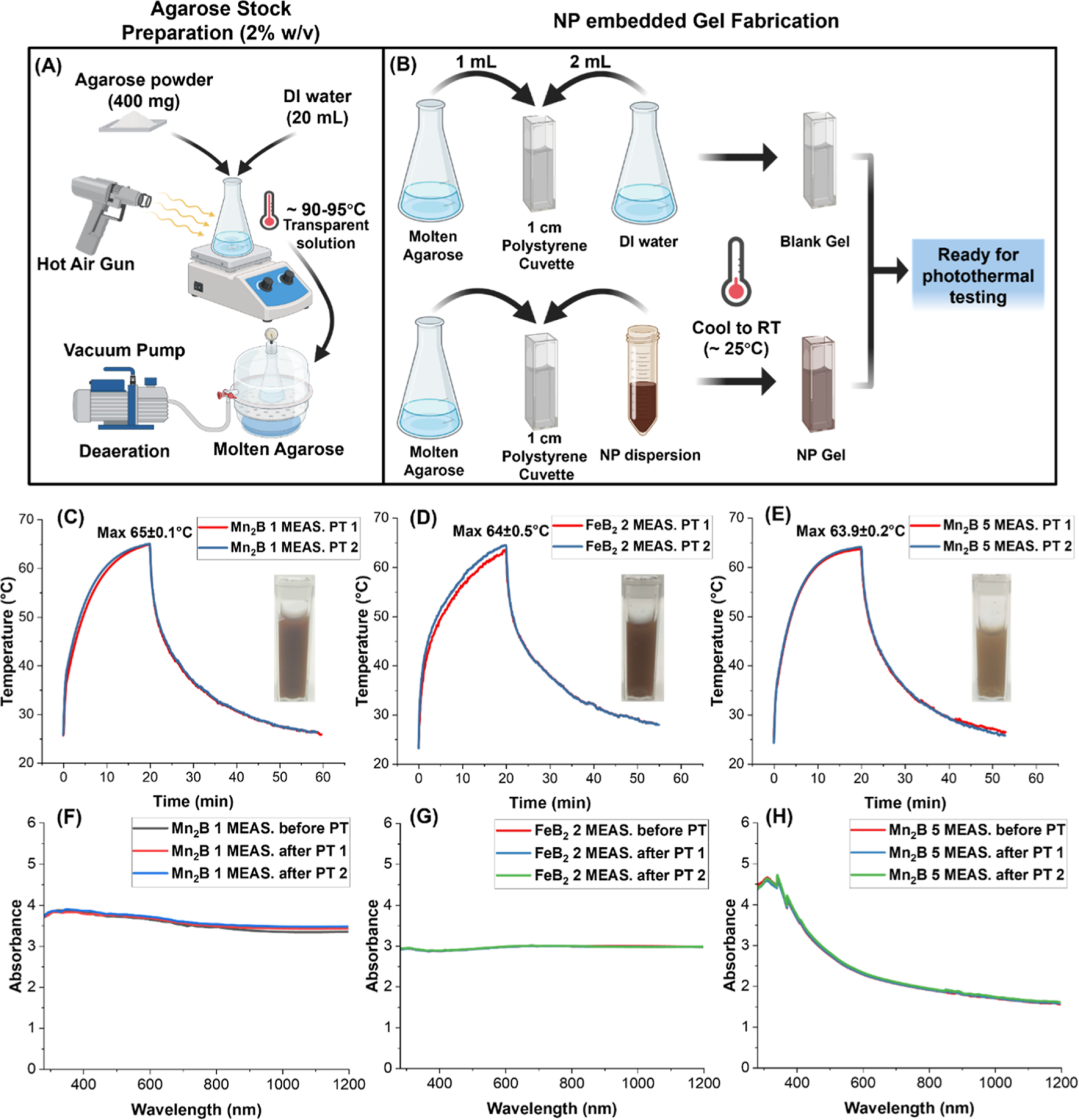
(A,B) Schematic workflow and PT experiment for temperature peak measurement in NP-gel samples, consisting of the preparation of agarose stock solution (A), and fabrication of gel-embedded NPs (B). (C–E) Heating–cooling profiles of NP-gel samples (Mn_2_B 1, FeB_2_ 2, and Mn_2_B 5) exhibiting the top three temperature peak metrics. (F–H) Optical absorbance spectra of the corresponding NP-gel samples before and after PT cycling, indicating photostability of the three samples.

Notably, Mn_2_B 1 NPs, synthesized in pure ethanol with 20 ns pulses, achieved the highest reached temperature peak of 65 ± 0.1 °C, coupled with the fastest temperature rise (Figure 3C). This performance is likely linked to its broadband absorption (Figure 2D), even though its concentration indicator was only moderate. Similarly, Mn_2_B 5 NP, synthesized with 200 ns pulses in ethanol–water, exhibited the third-highest peak temperature of 63.9 ± 0.2 °C (Figure 3E). Despite a narrower overall absorbance spectrum, this sample has a particularly large NIR absorption (Figure 2D). However, its concentration indicator is the lowest within the Mn_2_B group, indicating a significantly lower NP yield but with a more efficient light-to-heat conversion. All six samples in the Mn_2_B series show a negligible difference in the absorbance spectra before and after heating–cooling experiments, evidencing the photostability of the samples (Figures S5 and S6 in Supporting Information).

Although MnB_2_ samples showed high convolution area and concentration indicators, the temperature peaks were lower than in the Mn_2_B series. For instance, MnB_2_ 6 NPs reached a temperature peak of 63.6 ± 0.3 °C (Figure S7F), lower than the best Mn_2_B samples. This is likely due to particle aggregation and potentially higher scattering losses compared to Mn_2_B 1 NP. In addition, sample 3 synthesized in H_2_O with a 20 ns pulse duration, exhibited the highest peak temperature of 65 °C (Figure S7C) during the first PT experiment in this series. However, in the second trial, its maximum temperature decreased to 61 °C leading to the average peak of 63 ± 2 °C. This decline is likely attributed to a change in aggregation or oxidation during the PT experiment, which reduced true absorption and hindered stable PT conversion efficiency. The consistency between the two measurement cycles for six samples confirms their good stability as PT agents (Figure S8A–F).

Overall, a greater boron content correlates with enhanced structural stability and improved synthesis reproducibility during LAL, as reflected in both the FeB_2_ and MnB_2_ series, but the PT performance is more subtly influenced by aggregation and oxidation of the target compounds, resulting in a puzzle of difficult interpretation through a trivial empirical effort.

### 2.4 Regression Modeling of Synthesis toward Improvement of the NP Performance Metrics

A DOE-based regression model was constructed to investigate the correlation between experimental parameters (pulse duration (τ), solvent composition, and target composition) and the three NP performance metrics. The model development was carried out using the regression platform in the JMP Pro software.^46^ To ensure both accuracy and interpretability, the first step involved selecting appropriate model effects capable of capturing the trends in the synthesis data set. Specifically, both full factorial effects and second-order polynomial effects were considered to account for interaction and curvature in the relationships among the parameters. Subsequently, a stepwise regression procedure was applied, using the Bayesian Information Criterion (BIC) as the stopping rule.^47^ The BIC penalizes model complexity while rewarding goodness of fit, thereby preventing overfitting and ensuring that only the most relevant terms are retained. As a result, three analytical expressions (eqs 1–3) were identified, describing the dependence of the three NP performance metrics on the experimental parameters

1 $$\begin{array}{l} Y_{1}=62.462-0.0156\cdot Fe-0.0526\cdot Mn+3.167\cdot ethanol\\ +0.0163\cdot \tau \left(ns\right)-0.0051\cdot \left(ethanol\cdot \tau \left(ns\right)\right)-3.733\cdot \left(ethanol^{2}\right) \end{array}$$

2 $$\begin{array}{l} Y_{2}=1118.8918+0.5843\cdot Mn-6.6454\cdot B-49.6200\cdot ethanol\\ -0.7011\cdot \tau \left(ns\right)+0.1655\cdot \left(\left(Mn-38.184\right)\cdot \left(B-44.4\right)\right)\\ -0.0385\cdot \left(\left(Mn-38.184\right)\cdot \left(\tau \left(ns\right)-128\right)\right)+1.0416\cdot {\left(B-44.4\right)}^{2}\\ -561.4642\cdot {\left(ethanol-0.49333333\right)}^{2} \end{array}$$

3 $$\begin{array}{l} Y_{3}=\max(-3794.7076-137.0524\cdot Fe+141.2167\cdot B\\ -2395.2693\cdot ethanol-1.5993\cdot \tau \left(ns\right)\\ +0.5955\cdot \left(\left(Fe-17.316\right)\cdot \left(\tau -128\right)\right)\\ \begin{array}{l} +4.2431\cdot {\left(Fe-17.316\right)}^{2}+4.9701\cdot {\left(B-44.4\right)}^{2}\\ +7113.7223\cdot {\left(ethanol-0.49333333\right)}^{2},0) \end{array} \end{array}$$

where *Y*_1_ is the temperature peak (in °C), *Y*_2_ the convolution area (in W·m^–2^), *Y*_3_ is the NP concentration indicator (in mL·W·m^–2^). The Fe, Mn, and B contents in the DOE are expressed as the nominal atomic percentages (at %) in the Fe–Mn–B ablation target, normalized to a total of 100 at %. These variables therefore describe the target formulation used as synthesis input. The ethanol quantity is expressed as vol % in the water/ethanol liquid environment of the laser ablation synthesis.

The *R* ^2^ scores for *Y*_1_, *Y*_2_ and *Y*_3_ resulted, respectively, 0.9813, 0.9935, and 0.9635, indicating a good agreement between experimental and predicted value, as shown in Figure 4A–D. Despite these nonlinear models accurately fitted the LAL experimental data within the tested range, they are unbounded mathematical expressions which may also yield physically unrealistic negative predictions when extrapolated outside the range for experimental input values. To address this, we apply a postprocessing correction to eq 3, i.e., replacing negative values with zero, ensuring all outputs remain physically meaningful (≥0) while retaining the model’s predictive accuracy.

**Figure 4:**
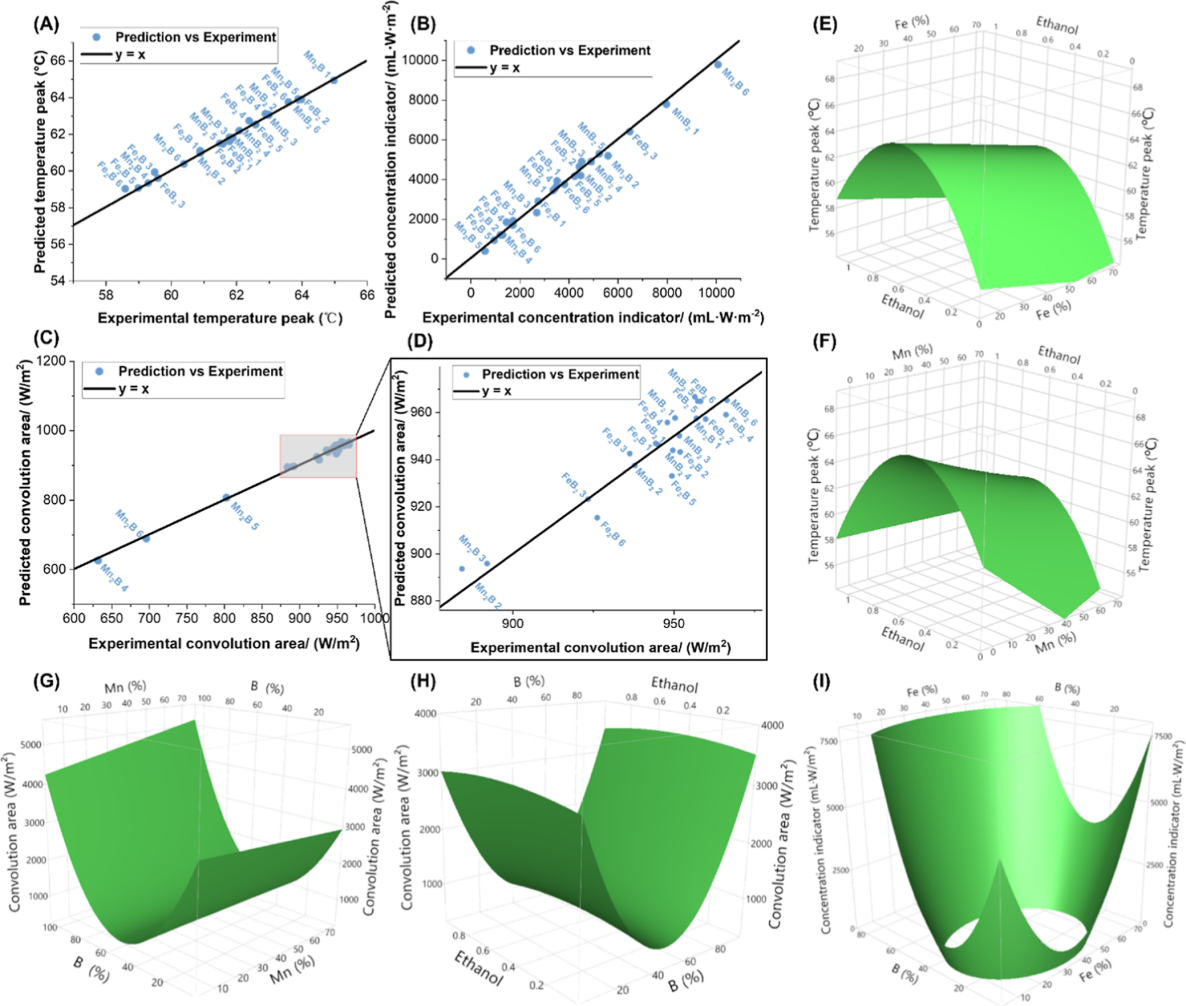
Predicted versus experimental values based on the three regression formulas for each NP performance metric: temperature peak (A), concentration indicator (B), convolution area (C,D). (D) Magnification of the inset in (C), showing the region with highest density of data points. (E) The nominal at % of Fe in target and ethanol in solvent; (F) the nominal at % of Mn in target and ethanol in solvent on the reached temperature peak of synthesized NPs. The coupling effect of (G) the nominal at % of Mn and B in target; (H) the nominal at % of B in target and ethanol in solvent on the convolution area between solar irradiation spectrum and the absorbance spectra of synthesized NPs. The coupling effect of (I) the nominal at % of Fe and B in target on the NP concentration indicator.

The above analytical expressions reveal parameter–metric interactions, as illustrated in Figures 4 and S9 in the Supporting Information for specific laser ablation and solvent conditions. In Figure 4E, when the ethanol content is below 50%, an increase in Fe proportion in the target leads to a progressive rise in the peak temperature. However, at 50% ethanol, further Fe addition reverses this trend. Overall, elevated Fe content is associated with lower peak temperature, also when pulse duration is changed (Figure S9A in the Supporting Information). A maximum in peak temperature is also observed for Mn contents of 10–20 at % combined with ∼50% ethanol (Figure 4F). The Mn content and pulse duration interaction exhibits a similar pattern (Figure S9B in the Supporting Information), yet the heating effect is more significant for 200 ns pulses than for 20 ns. These trends highlight the critical role of composition-dependent optical and thermal properties in combination with solvent properties and pulse duration.

The two-way interactions for the convolution area metric also indicate a consistent dependence on the composition of target and liquid environment (Figures 4G,H and S9C–F in the Supporting Information). Varying Mn and B in the target produces a saddle surface (Figure 4G), since the metric is lowest at low–moderate B with low Mn, and increases toward the high-B and higher-Mn edges. This trend is consistent with B-rich compositions promoting NIR extinction, with moderate Mn content contributing to optical absorption in the visible range, although excessive Mn ratios elicit oxidation and degradation of the broadband optical properties. Mapping Mn against ethanol fraction shows a broad ridge centered near ∼50% ethanol and Mn ≈ 10–30 at % (Figure S9D in the Supporting Information). For B versus pulse duration (Figure S9C in the Supporting Information), a U-shaped dependence on B is evident with a trough at low-intermediate B. B versus ethanol displays a similar mixed-solvent ridge near ∼50% ethanol together with a U-like dependence on B (Figure 4H), reflecting the balance between rapid quenching (water-rich environment) and organic passivation (ethanol-rich environment). The Mn–pulse map shows a pronounced valley at low Mn with long pulses (consistent with Mn-oxidation and reduced absorptivity) and a monotonic rise with Mn under 200 ns (Figure S9E in the Supporting Information); at 20 ns the surface is flatter, suggesting weaker pulse-length sensitivity at low Mn. Ethanol versus pulse duration (Figure S9F in the Supporting Information) yields a dome-like surface with a maximum near ∼50% ethanol; pulse-length effects are modest in neat solvents but become more favorable for 200 ns in the mixed solvent. Taken together, these maps indicate that mixed ethanol–water (∼50%) and B-rich or moderately Mn-containing targets promote broad, solar-weighted absorption; regions with low Mn with long pulses constitute minima of the metrics, likely due to Mn-oxidation and limited broadband spectrum formation.

The two-way interaction effects between target composition, solvent composition, and laser pulse parameters on the NP concentration indicator are shown in Figures 4I and S9G-I in the Supporting Information The Fe–B interaction exhibits a broad minimum at intermediate B with low Fe and increases sharply toward high B or high Fe content (Figure 4I). The trough at intermediate B and low Fe reflects insufficient particle production. In the Fe-ethanol map (Figure S9G in the Supporting Information), the lowest concentrations occur near moderate Fe, while high Fe with high ethanol, or low Fe with low ethanol, yield elevated particle concentrations. For B versus pulse duration (Figure S9H in the Supporting Information), the concentration indicator increases monotonically at high B content, while remaining low for low B regardless of pulse duration. The B-ethanol map (Figure S9I in the Supporting Information) shows a similar U-shaped dependence on B and ethanol, with minima at intermediate B and ethanol ∼50%, and maxima at B-rich compositions in any solvent. Collectively, these patterns indicate that high NP concentrations are favored by either Fe-rich or B-rich targets, while intermediate compositions and mixed solvents usually suppress LAL yield. These trends also rationalize the initial choice of the Fe–Mn–B family. Boron promotes broadband absorption and structural stability, whereas Fe and Mn expand the accessible photothermal and structural design space. At the same time, the composition of target and solvent must be balanced to avoid excessive oxidation and yield penalties.

Based on a comprehensive analysis of the two-way interaction terms, the DOE model was further interrogated in JMP using desirability profiling (Derringer–Suich) to jointly optimize the three NP performance metrics. In this procedure, each response was transformed into an individual desirability function and combined into an overall desirability objective, and the optimal synthesis conditions were identified by maximizing this overall desirability. The resulting solutions were reported as points on the multivariate desirability Pareto Frontier (i.e., nondominated trade-offs in desirability space) for the three NP performance metrics within the experimentally sampled parameter domain.^48,49^ Notably, the synergistic effect of different experimental parameters needs to be considered for simultaneously maximizing the three NP metrics. Therefore, the optimal condition identified in this study, which are Fe_10_Mn_24_B_66_ target (composition expressed in at %), 1:1 ethanol–water solvent mixture, and pulse duration of 25 ns, represents the intersection of favorable trends observed across the three performance metrics. The high B content enhances broadband NIR absorption and stabilizes the NPs, thereby promoting both spectral overlap and particle yield, as Figure S9C in the Supporting Information shows the U-shaped B dependence with a rising branch at high B, and Figure S9H–I in the Supporting Information illustrates that B-rich targets improve concentration under both pulse and solvent conditions. A limited Fe fraction (10 at %) does not significantly affect peak temperature (Figures 4E and S9A in the Supporting Information), while simultaneously retaining NP yield, as Figures 4I and S9G in the Supporting Information show that Fe enhances yield, though excessive Fe in mixed solvents can introduce concentration troughs. The moderate Mn level, although slightly above the 10–20 at % range associated with maximum peak temperature, remains effective under short-pulse and mixed-solvent conditions by mitigating Mn-oxidation, as Figures 4F and S9B in the Supporting Information highlight the 10–20 at % optimal Mn window, and Figure S9E in the Supporting Information demonstrates low-Mn valleys under long pulses, while Figure S9D in the Supporting Information indicates solvent-Mn coupling favoring moderate Mn in mixed media. The mixed ethanol–water solvent is associated with broad absorption spectra, because both Figures 4H and S9D in the Supporting Information consistently show a ridge near ∼50% ethanol. Figure S9G,I in the Supporting Information confirm that solvent–composition interactions produce maxima in water-rich or ethanol-rich regimes but can be balanced at 1:1 when combined with high B and short pulses. Finally, a 25 ns pulse duration offers sufficient yield while avoiding the excessive oxidative effects characteristic of longer pulses, as Figure S9B,D in the Supporting Information demonstrate suppression of peak temperature for high Fe content at long pulses, Figure S9C,F in the Supporting Information show enhanced absorption at midshort pulses, and Figure S9H in the Supporting Information confirms that NP concentration monotonically rises at high B at any pulse duration (20–200 ns), meaning that 25 ns achieves near-optimal yield without requiring 200 ns.

### 2.5 Final Optimization of NP Synthesis

#### 2.5.1 Experimental Optimization of the Predicted Best NP Synthesis Conditions

The DOE was performed under moderate LAL pulse energy (0.28 mJ/pulse) but high repetition rate (50 kHz) to ensure optimal NPs productivity, thus enabling a rapid and statistically reliable prescreening of target and solvent composition. However, literature indicates high-energy (>10 mJ/pulse) and low-repetition-rate (50 Hz) laser pulses in acetone, as valid condition for the synthesis of boron-based multielement nanostructures.^50,51^ Pulse energies above 10 mJ/pulse promote alloying and heterostructure formation from mixed-powder targets, while acetone limits oxidation and contributes to carbon-containing nanostructure formation.^52–55^ These conditions are not practically suited to the generation of an extensive DOE matrix, as the lower repetition rate entails substantially longer synthesis times and an approximately 20-fold reduction in productivity, despite the higher pulse energy. Accordingly, the DOE was not conceived as the final optimization of the synthetic pathway leading to the best-performing Fe–Mn–B nanoparticles. Instead, it served as a statistically guided, high-throughput screening step to identify a robust compositional starting point. This DOE-derived formulation was then subjected to a focused refinement of the LAL parameters, including a limited number of targeted experiments under longer synthesis conditions, to conclusively identify the conditions leading to the best-performing Fe–Mn–B nanoparticles.

In particular, four different LAL conditions were tested, while maintaining the same target composition of Fe_10_Mn_24_B_66_ (recommended by DOE) and laser wavelength of 1064 nm: (a) 1:1 ethanol–water mixture, synthesis duration 5 min, pulse duration 25 ns, 0.28 mJ/pulse, repetition rate 50 kHz (best DOE prediction); (b) acetone, synthesis duration 5 min, pulse duration 25 ns, 0.28 mJ/pulse, repetition rate 50 kHz; (c) 1:1 ethanol–water mixture, synthesis duration 90 min, 50 mJ/pulse, repetition rate 50 Hz (the highest available for the high-energy pulsed laser); (d) acetone, synthesis duration 90 min, 50 mJ/pulse, repetition rate 50 Hz. In this way, the effect on the performance metric of the two parameters known in literature for favoring the formation of multielement NPs, that are the use of acetone and high-energy laser pulses, can be comparatively verified against the DOE results.

Indeed, among the four synthesis conditions, sample d exhibits the broadest and stable absorbance profile (Figure 5A), with the largest convolution area with the solar spectrum of 996 W/m^2^ (Figure 5B) and the highest reached temperature peak of 70.7 ± 0.8 °C (Figure 5C,D). The concentration indicator (9518 mL·W·m^–2^, Figure 5B) is also larger than in sample c. However, this last metric cannot be directly compared to the samples obtained with the low pulse energy laser source, which has a higher throughput because of the higher repetition rate and overall laser power, reaching comparable concentration indicators after only 5 min versus the 90 min required for samples c and d. On the other hand, the higher pulse energy and use of acetone favors the formation of compositionally heterogeneous NPs containing mixtures of metal and oxide phases with abundant interfacial defects and optimal stability, as required for the maximization of the metrics relevant to ISSG.^35,56–60^ For synthesis c in 1:1 ethanol–water mixture, the convolution area is 926.95 W/m^2^ (Figure 5B) and the temperature peak 65 ± 0.1 °C (Figures 5D and S10C), accompanied by a concentration indicator of 8277 mL·W·m^–2^, all lower than in sample d. By comparison, samples a and b show less broadened absorption (Figure 5A). Consequently, their convolution area metrics (Figure 5B) and temperature peaks (65.9 ± 0.1 °C and 64.5 ± 0.2 °C, respectively, see Figures 5D and S10A,B) are lower than sample d. While the lower concentration indicator (5912 mL·W·m^–2^ and 5321 mL·W·m^–2^ for, respectively, sample a and b) compared to samples c and d arises from the shorter (5 min) irradiation time, this metric indicates better productivity in the ethanol/water mixture than in acetone for the low-energy high-repetition rate laser source. Moreover, the temperature deviation and absorbance spectra before and after two heating–cooling experiments (Figure S11 in the Supporting Information) confirm the PT stability of all the 4 samples.

**Figure 5:**
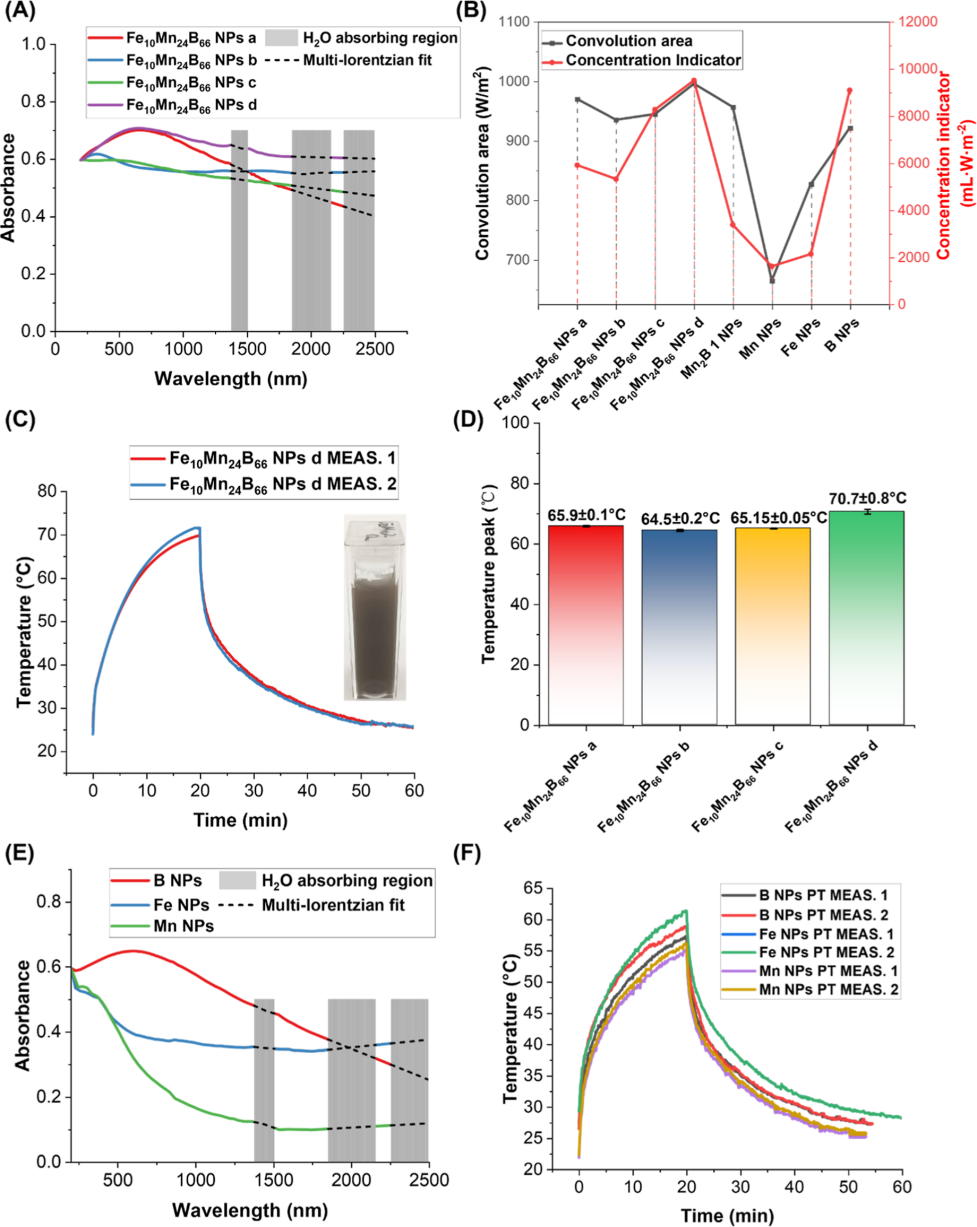
(A) The absorbance spectra of Fe–Mn–B NPs sample a—synthesized in 1:1 ethanol-to-water mixture with 0.28 mJ/pulse, 50 kHz laser pulses; b— synthesized in acetone with 0.28 mJ/pulse, 50 kHz laser pulses; c—synthesized in 1:1 ethanol-to-water mixture with 50 mJ/pulse, 50 Hz laser pulses; d—synthesized in acetone with 50 mJ/pulse, 50 Hz laser pulses. (B) The convolution area and concentration indicator of Fe–Mn–B NPs a–d, Fe, B and Mn NPs. (C) Heating–cooling curves of Fe–Mn–B NPs d. (D) The comparison of temperature peak of Fe–Mn–B NPs a–d under the same irradiation conditions. (E) The absorbance spectra of Fe, B and Mn synthesized in 1:1 ethanol-to-water mixture synthesized in pure ethanol by with 0.28 mJ/pulse, 50 kHz laser pulses. (F) Heating–cooling curves of Fe, B and Mn NPs.

To validate the necessity of multielement NPs, the single-element Fe, B, and Mn NPs were also synthesized from the corresponding pure-element targets adopting the same experimental conditions obtained from the DOE prediction, as for synthesis a. As shown in Figure 5E, pure B NPs exhibit a moderate but relatively uniform broadband absorption across the visible-NIR range, with a peak in the visible region. This absorption is accompanied by a temperature peak of 58.1 ± 0.8 °C (Figure 5F) and a high synthesis yield of 9100 mL·W·m^–2^ (Figure 5B). In contrast, Fe and Mn NPs display absorbance profiles peaked in the UV, with a rapid decay in the visible-NIR region (Figure 5E). This behavior originates from their chemical reactivity during LAL, since both Fe and Mn rapidly oxidize and aggregate, forming oxide shells and agglomerates that suppress free-carrier absorption and increase light scattering at the expense of real absorption.^61^ Consequently, the spectra of Fe and Mn NPs lack stable broadband absorption, and the convolution area is low (respectively, 827 W·m^–2^ and 665 W·m^–2^). The concentration indicators of 2130 mL·W·m^–2^ (Fe) and 1609 mL·W·m^–2^ (Mn) are also lower than the multielement NPs (Figure 5B). Although B nanostructures provide the most contribution to productivity and PT properties, the comparison with the NPs from the multielement target Fe_10_Mn_24_B_66_ shows that the latter exhibit markedly broader and more stable absorption spectra (Figure 5A,B).

These observations confirm that the DOE-predicted synthesis conditions for the Fe_10_Mn_24_B_66_ target (synthesis a) provide enhanced performance metrics compared to NPs achieved from a single-element target, as well as to the best binary NPs, in particular the sample Mn_2_B 1 in the starting data set. Furthermore, the use of high-energy laser pulses and acetone (synthesis d) offers the most favorable conditions for producing a multielement NP system with superior PT performance.

#### 2.5.2 Structural Analysis of Optimized Nanoparticles

The chemical environment during pulsed laser ablation in liquids fundamentally dictates the nanoarchitecture, phase composition, and resultant functional properties of multicomponent NPs. Here, to further investigate the nanostructure and elemental composition of the best-performing sample, Fe–Mn–B d, the NPs were immobilized onto a commercial cellulose acetate (CA) membrane, which provided a convenient and mechanically stable support for SEM, EDS and XRD characterization, as well as for subsequent solar steam generation tests (see Methods for details of the membrane preparation). Herein, the SEM analysis reveals a heterostructure comprising crystalline Fe and Mn oxide particles dispersed in a B matrix (Figure 6A–I). The Fe and Mn oxide particles appear in top-view (Figure 6A–C and cross-sectional Figure 6G–I) SEM images as small, bright, roughly spherical nanocrystals (typically 30–80 nm in diameter) embedded in a lower electron dense matrix. The EDS elemental maps illustrate this architecture (Figure 6D–F) as made of Fe and Mn localized in discrete nuclei, while B forms a continuous, diffuse background. Also, O enrichment is preferentially associated with Mn-rich regions, with lower intensity on Fe-rich regions (Figure 6F). The diffuse carbon signal detected across the entire sample (Figure 6E,H), most likely originating from the decomposition of acetone under intense laser irradiation, points to the possible formation of an external carbon shell around the NPs.

**Figure 6:**
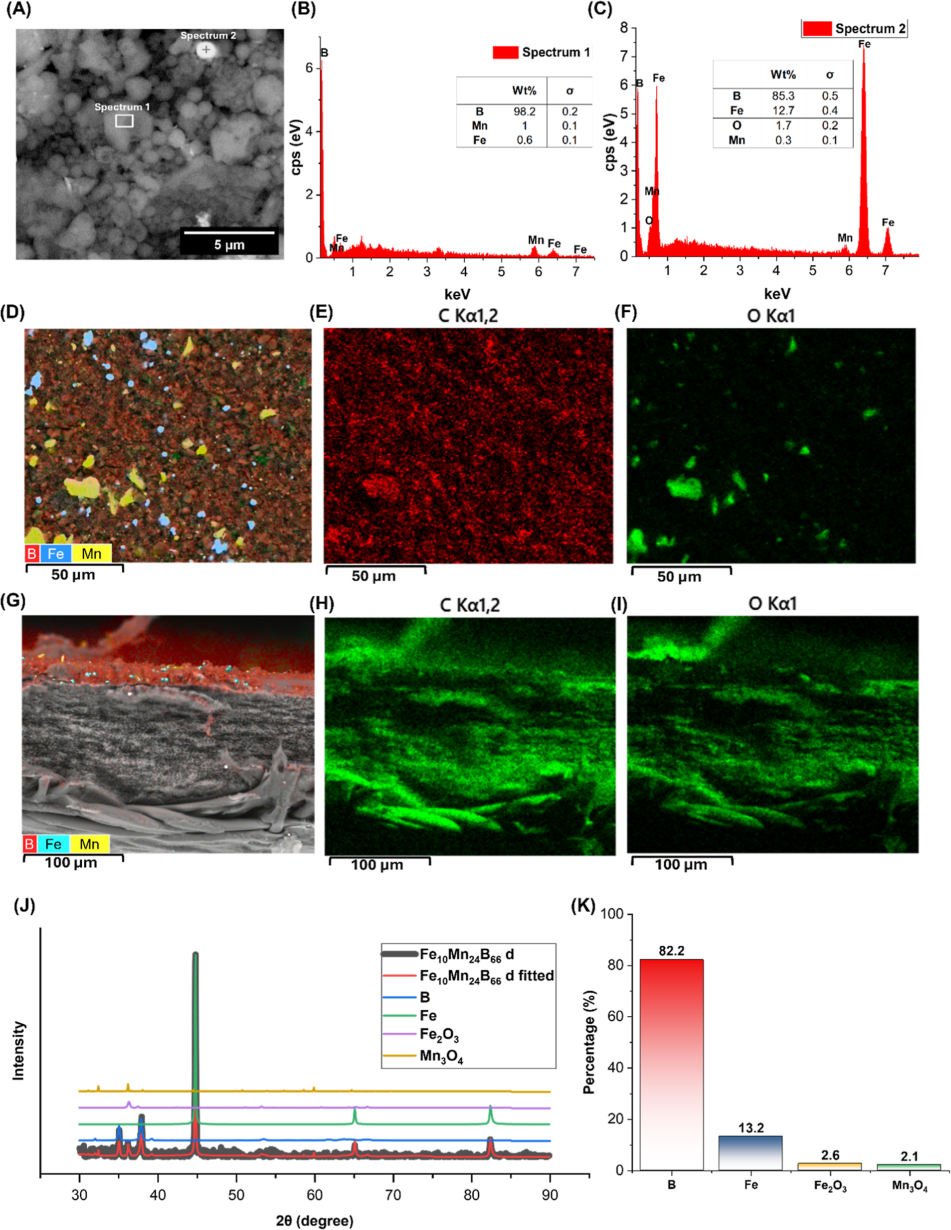
(A) The SEM morphology analysis of Fe–Mn–B d NPs. (B,C) The point element analysis of spectra 1–2 in the SEM image (A). (D) EDS elemental mapping of Fe–Mn–B d NPs, showing B in red, Fe in blue, and Mn in yellow. (E) Broad distribution of C across the entire region is shown in (D). (F) O enrichment is preferentially located on Mn-rich regions, with lower intensity on Fe-rich regions in (D). (G) Cross-sectional SEM morphology of Fe–Mn–B d NPs deposited on a cellulose filter, with elemental mapping of B (red), Fe (cyan), and Mn (yellow). (H) Broad distribution of C across the Fe–Mn–B region in (G). (I) Scattered distribution of O within the Fe–Mn–B cross-section in (G). (J) The XRD analysis and Rietveld refinement fitting of Fe–Mn–B NPs d. (K) The crystalline phase composition of Fe–Mn–B NPs d.

The elemental EDS quantification for this complex structure (Figure 6B,C) poses some threats due to the peculiarity of B characteristic X-ray emission. The consistently high B signal in EDS analysis does not represent the true bulk stoichiometry of the sample due to two key factors. First, the proximate energy peaks of B Kα (0.183 keV) and C Kα (0.277 keV) cause significant signal overlap in EDS detectors, leading to an artificial overestimation of the B content at the expense of C, which cannot be corrected. Second, as EDS is inherently more sensitive to the surface of a sample, its analysis of a surface segregated architecture will predominantly probe the outer matrix rich in B and C, underestimating the Fe and Mn component. Therefore, the measured composition is a surface-weighted average, not the absolute elemental ratio. These considerations are substantiated by cross-sectional element mapping EDS analysis (Figure S12A,B in the Supporting Information), with an increase of Fe and Mn (Fe 8.7 wt % and Mn 6.3 wt %) within the cores on its cross-section area compared to the surface element mapping EDS analysis (Fe 0.5 wt % and Mn 0.6 wt %).

Another bulk estimation of sample crystalline composition can be achieved through Rietveld refinement of the XRD pattern (Figure 6J–K). Here, XRD analysis revealed that the Fe–Mn–B NPs (sample d) consist of B (82.2 wt %), Fe (13.2 wt %), Fe_2_O_3_ (2.6 wt %), and Mn_3_O_4_ (2.1 wt %). This bulk crystalline structure is key to understanding the EDS analysis. The XRD analysis has not identified carbides, although the elemental EDS mapping did not spatially resolve the B-matrix from the C component. Therefore, the chemical pathway of solvent pyrolysis at the hot plasma–liquid interface evolves toward deposition of the carbonaceous species onto the already-formed NP surfaces, thus constituting the outermost layer.

X-ray photoelectron spectroscopy was performed on the Fe–Mn–B d NPs to analyze the valence states of Fe and Mn and chemical states of B as well as the nature of the carbon incorporated in the sample. The B 1s spectrum (Figure S13A in Supporting Information) has a well-defined peak centered at 187.7 eV of binding energy (BE), corresponds to metallic boron. The C 1s spectrum (Figure S13B in Supporting Information) has a dominating component at 284.8 eV, typical of aliphatic hydrocarbon, but also a minor component at higher BE (287.0 eV) assigned to oxidized carbon (C–O). This is compatible with the presence of amorphous carbon derived from solvent pyrolysis during LAL. Regarding the Mn 2p (Figure S13C in Supporting Information) and Fe 2p (Figure S13D in Supporting Information) spectra, both are fitted with a single component corresponding to, respectively, MnO and Fe_2_O_3_. Therefore, surface Mn and Fe atoms have higher oxidation state than that expected, on average, from the crystalline phases identified by the XRD analysis (Fe, Fe_2_O_3_ and Mn_3_O_4_), whereas B surface atoms only are in the metallic state, which is compatible with the presence of a protective shell of amorphous hydrocarbon.

This hypothesis was further substantiated at the single-boron-nanostructure level by high-resolution transmission electron microscopy analysis (HRTEM), as well as by scanning-TEM imaging and analytical techniques (Figure S14 in the Supporting Information). HRTEM images (Figure S14A in the Supporting Information) show that the boron NPs are surrounded by an amorphous shell or matrix, thereby excluding the presence of graphitic carbon. STEM analysis of B NPs of different sizes (Figure S14B,C in the Supporting Information) shows that carbon is present around the B NPs and that this carbon-based material also contains oxygen, as indicated by XPS. The backscattered electron (BSE) and secondary electron (SE) STEM images collected from the same group of NPs indicate that the coating is three-dimensional and extends over the boron NPs. This carbon coating is not observed in the pristine powder used for the LAL target (Figure S15 in the Supporting Information), indicating that it is generated during the laser ablation process.

An external amorphous carbon layer is a known and highly efficient broadband absorber. Thus, the resulting Fe/Mn/B@C architecture achieved in acetone with high-energy laser pulses may explain the improved PT performance (Figure 5C) compared to the other samples.

Indeed, additional useful insights on the NPs formation are obtained by the analysis of the Fe–Mn–B sample c, obtained in the more oxidizing ethanol–water environment (Figure S16 in Supporting Information). In this case, the phase-separated structure is more evident than in sample d, because several large (100–300 nm), agglomerated, and irregularly shaped Fe and Mn oxide particles are dispersed in the B-rich phase (Figure S16A–F in the Supporting Information). EDS point analyses on the bright domains, both on the surface (Figure S16A–C in the Supporting Information) and deep within the cross-section (Figure S16G–I in the Supporting Information), confirm they are significantly enriched in Fe (∼30–50 wt %), Mn (∼5–10 wt %), and oxygen (∼8–10 wt %). The high oxygen content is visually corroborated by detailed EDS mapping (Figure S16B,C,G in the Supporting Information), which shows a strong spatial correlation between the O, Mn and Fe signals. Together, these findings confirm the in situ oxidation of the metallic components, aligning with XRD analysis (Figure S16J–K in the Supporting Information), which reveals the presence of B (80.6 wt %), Fe (13.8 wt %), MnO (2.7 wt %) and Mn_3_O_4_ (2.9 wt %) and indicates the prevalence of Mn oxides. The presence of high oxygen content deep within the SEM cross-section confirms that oxidation is a bulk phenomenon, meaning the metallic domains are oxidized throughout their entire volume, not just on their surface. This stands in sharp contrast to the negligible oxidation observed in the acetone synthesis. The diverse nanoarchitecture is attributed to the abundant availability of reactive oxygen species from water molecules. The difference in structure and composition between samples c and d directly translates to the worse functional properties of the former.

Notably, the Mn content estimated from EDS and XRD analysis in the Fe–Mn–B c and d NPs is lower than in the ablated target. This loss of Mn could be attributed to either a lower ablation rate compared to Fe and B grains, or higher solubility in the liquid phase of Mn, resulting in removal during the centrifugation and washing steps, thereby causing a deviation of the final elemental composition from the target stoichiometry.

Overall, we demonstrate that ablating a single Fe_10_Mn_24_B_66_ metal target under identical laser parameters but switching the solvent from pure acetone to a 1:1 ethanol–water mixture, alters the NP formation pathway and yields two distinct classes of heterostructured materials with different PT properties.

### 2.6 Solar Steam Generation and Water Purification with the Optimized Fe–Mn–B NPs

#### 2.6.1 Solar Water Evaporation

To translate the positive photothermal properties into an application-relevant benchmark, we evaluated the Fe–Mn–B d NPs in a proof-of-concept interfacial solar steam generation setup. Based on the previously prepared NP coated CA film, the solar water evaporation experiments were performed in a beaker containing 50 mL of deionized water and a polystyrene floating device for holding the cellulose substrate (Figure 7A). The beaker was positioned under the outlet of the solar simulator equipped with a 90° beam-turning collimator. Under these conditions, the irradiation corresponded to an effective 3.5-sun intensity.

**Figure 7:**
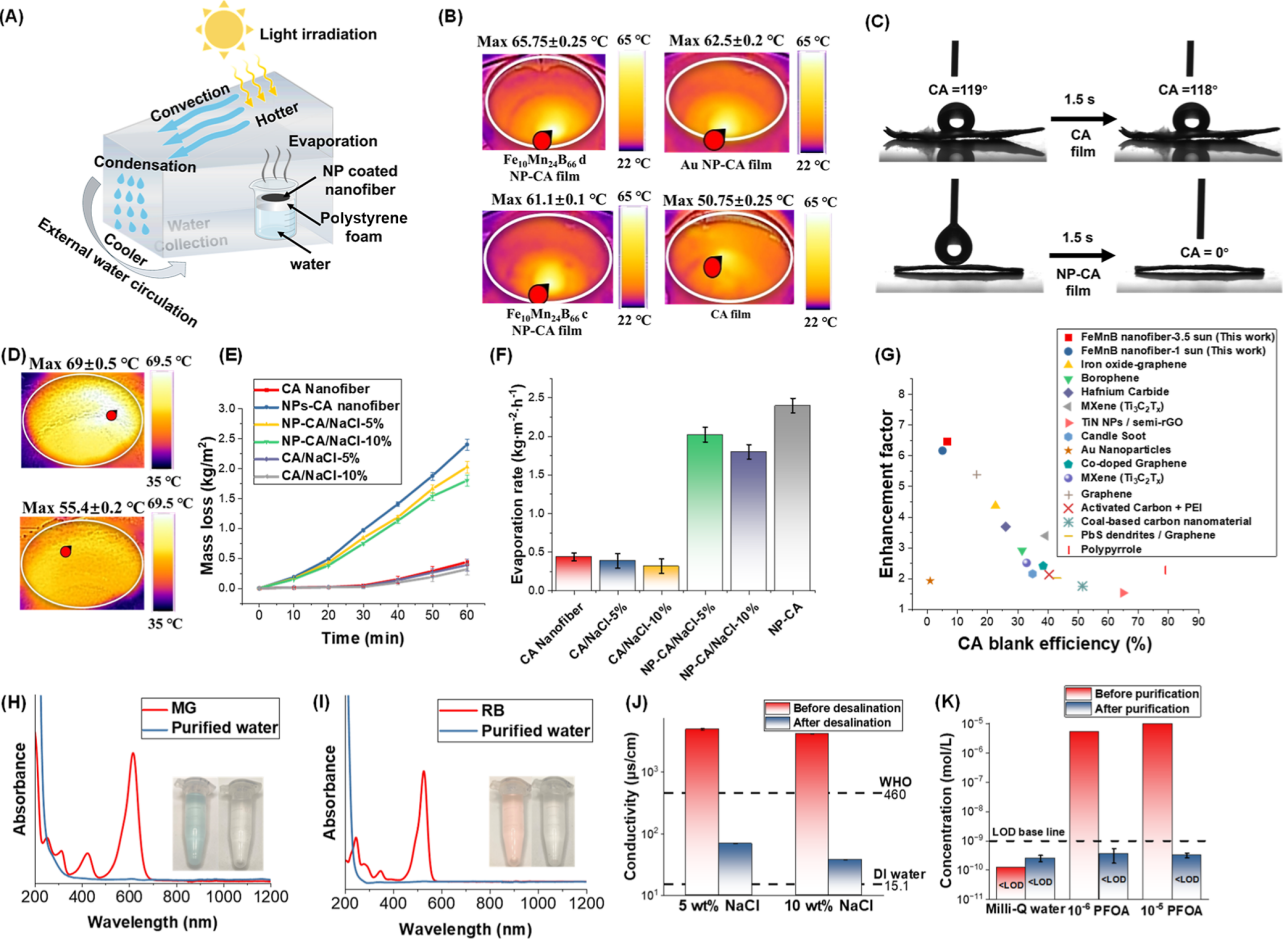
(A) The sketch of the ISSG system. (B) The reached peak temperature of Fe–Mn–B d NPs, Au NP benchmark (nanocorals^62^), Fe–Mn–B c NPs, and blank CA substrate under the solar simulator with 3.5-sun intensity. (C) Water contact angle change over time for blank CA nanofiber and Fe–Mn–B d NP nanofiber. (D) The reached peak temperature of Fe–Mn–B d NP nanofiber evaporator and blank CA nanofiber under the solar simulator with 3.5-sun intensity. (E,F) The mass loss versus irradiation time and the evaporation rate of blank CA nanofiber, Fe–Mn–B d NP nanofiber in the deionized (DI) water, 5 and 10 wt % NaCl aqueous solution under sunlight simulator with 3.5-sun intensity. (G) The comparison of enhancement factor versus blank CA nanofiber efficiency from the literature using CA nanofiber substrate for ISSG application. Dye wastewater treatment of the Fe–Mn–B d NP nanofiber evaporator. UV–vis spectra of MG (H) and RB (I) aqueous solution before and after purification, and the corresponding digital photographs. (J) The conductivity of 5 and 10 wt % NaCl aqueous solutions before and after desalination. (K) The concentration of PFOA in the blank (Milli-Q water) and spiked PFOA 1.0 × 10^–6^ and 1.0 × 10^–5^ mol·L^–1^ solutions, before and after purification.

For benchmarking, broadband-absorbing Au nanocorals (Au NPs) from our previous work^62^ were deposited on the same CA membrane support and tested under the same solar simulator, effective irradiation intensity (3.5 suns), and floating-device configuration used for the Fe–Mn–B coatings. The Au NPs benchmark had an average longitudinal length of 80 ± 38 nm and transversal length of 24 ± 9 nm, with near unity solar-weighted absorption coefficient,^62^ and was deposited from an aqueous dispersion with an Au concentration of 0.355 ± 0.013 mg·mL^–1^. As shown in Figures 7B and S17 in the Supporting Information, the mass loss of water increased steadily with irradiation time, with the Fe–Mn–B d NPs exhibiting the highest evaporation rate, followed by the Fe–Mn–B c NPs, while Au NPs and the bare CA control displayed comparatively lower performance. The corresponding infrared thermal images further confirm this trend: the surface temperature of the Fe–Mn–B d coated floater reached 65.75 ± 0.25 °C under 3.5-sun irradiation, which is higher than that of Au NPs (62.5 ± 0.2 °C) and Fe–Mn–B c NPs (61.1 ± 0.1 °C), all of which shows the notable temperature elevation, much higher than that of the blank CA film (50.75 ± 0.25 °C).

Compared to the background evaporation rate (0.69 ± 0.05 kg·m^–2^·h^–1^) due to the absorption of water and cellulose in the solar spectrum, the substrate coated with Fe–Mn–B d NPs provided an increment of +248% in steam formation (Figure S17 in the Supporting Information), corresponding to a steam generation rate of 2.40 ± 0.05 kg·m^–2^·h^–1^. Despite the simplicity of this solar steam generation device, the final result is comparable to other devices with more complex designs and production protocols.^63–66^ The benefit in the use of Fe/Mn/B@C architecture resulted in a 102.3% and 39.3% higher steam formation rate in comparison to the Fe–Mn–B c NPs (1.187 ± 0.03 kg·m^–2^·h^–1^) and Au NPs (1.725 ± 0.07 kg·m^–2^·h^–1^), respectively.

To further advance the solar steam generation and water purification experiments, CA electrospun nanofiber membranes were employed as an alternative to the previously used commercial CA films. Compared with the limited filtration area of commercial CA membranes, CA electrospun nanofiber matt provide a tunable and significantly larger surface area, while offering great porosity, permeability, and the potential to improve the light-harvesting capability of nanocomposites.^67,68^ As shown in Figures 7C and S18 in the Supporting Information, the pristine CA nanofiber membrane is intrinsically hydrophobic, exhibiting a stable water contact angle of ∼119 ± 1° over the first ∼1.5 s. In sharp contrast, after NP coating (NP–CA nanofiber), the apparent contact angle rapidly decays to ∼0 ± 1° within ∼1.5 s, indicating complete wetting and fast liquid imbibition into the porous network. This wettability switch is critical for ISSG, because hydrophilic and interconnected pores enable sustained capillary pumping, mitigate local dry-out, and promote the formation of a thin and continuously replenished interfacial water layer, all things that are widely recognized as key enablers for efficient heat localization and high evaporation flux.^10^

Under identical illumination of 3.5-sun intensity, the area-normalized evaporation rates of the cellulose filter-based evaporator (15 mm) and nanofiber-based evaporator (35 mm) systems are 2.40 ± 0.05 kg·m^–2^·h^–1^ and 2.40 ± 0.10 kg·m^–2^·h^–1^, respectively, indicating that the performance was retained on the >5× larger area. The 3.5-sun measurements are retained here mainly to benchmark the optimized Fe–Mn–B coating against the Au reference and to compare the commercial CA and nanofiber membrane formats at the level of a proof-of-concept device. The reached temperature peak of the nanofiber-based evaporator is moderately higher than that of the cellulose filter-based evaporator (Figure 7D). This indicates that the intrinsic PT conversion efficiency and water transport capacity of the coated nanostructures remain essentially unchanged across the two system sizes. This retention of area-normalized evaporation rate upon >5× increase in active area suggests that the advantage of the optimized Fe–Mn–B coating is preserved within the present scale-up window. This proof-of-concept scale-up test is encouraging for larger-scale applications, although further development will require addressing several challenges associated with coupled transport phenomena, including vapor diffusion and removal, water supply, thermal losses, and, for saline feeds, salt accumulation management.^29,30,73,74^ The area-normalized evaporation rates of the nanofiber-based evaporator and the blank CA nanofiber membrane under 1-sun intensity are 0.56 ± 0.02 kg·m^–2^·h^–1^ and 0.160 ± 0.007 kg·m^–2^·h^–1^ (Figure S19 of the Supporting Information). Using eq 4 in the Methods section and the parameters reported in Table S2 of the Supporting Information, the corresponding solar-to-vapor efficiencies are 43.0% for the NP-coated cellulose-film evaporator under 3.5 suns and 31.3% for the NP-coated electrospun nanofiber evaporator under 1 sun. The moderate 1-sun evaporation rate is reasonable for a proof-of-concept 2D membrane architecture. Recent literature has identified a commonly cited upper bound of about 1.47 kg m^–2^·h^–1^ for two-dimensional absorber surfaces under 1 sun, while a comparative study of six model materials reported 1.21 kg m^–2^·h^–1^ as the best-performing value and cautioned that small-area 2D measurements can overestimate apparent performance.^71,72^ Indeed, our experimental 2D configuration was selected for a reliable and rapid evaluation of the enhancement over the blank substrate, as well as for the reproducible assessment of the DOE-guided LAL route to the optimization of an earth-abundant Fe–Mn–B photothermal membrane platform. Therefore, further improvements may be expected from architectural and transport-level optimization, including 3D evaporator design, improved thermal management, and enhanced water/salt transport.

To evaluate desalination-relevant operation, the NP–CA nanofiber evaporator was further tested using brines of 5 and 10 wt % NaCl (Figure 7E,F). According to the literature, increasing the salt concentration leads to a reduction in mass loss and evaporation rate relative to DI water.^73–76^ Indeed, the 10 wt % NaCl (1.80 kg·m^–2^·h^–1^) feed shows a consistently lower evaporation flux than 5 wt % (2.02 kg·m^–2^·h^–1^) under identical irradiation. This trend is expected because higher salinity decreases water activity, thereby lowering the equilibrium vapor pressure and the chemical potential driving force for phase change, and increases solution viscosity/density, which together can hinder capillary replenishment and slow down interfacial water transport in porous evaporators.^69,70,77^ Additional repeated-cycle and continuous-operation tests under 10 wt % NaCl at both 1 sun and 3.5 suns further confirmed the operational durability of the NP–CA membrane (Figures S20–S23). The membrane used in these durability tests had been stored for about 4 months before re-evaluation. The results show no obvious performance degradation. Under 1 sun, the first-hour evaporation rate in 10 wt % NaCl was 0.518 kg·m^–2^·h^–1^, close to the previously reported 0.56 ± 0.02 kg·m^–2^·h^–1^ under 1 sun in pure water, while under 3.5 suns the 3 h average evaporation rate in 10 wt % NaCl was 1.785 kg·m^–2^·h^–1^, close to the ∼1.8 kg·m^–2^·h^–1^ value previously reported under the same salinity and irradiation conditions. After the initial stage of the additional continuous-operation tests, required to establish thermal equilibration of the membrane-water-container system under continuous irradiation, a higher quasi-steady evaporation regime is established. The repeated-cycle 1 h tests also showed only small cycle-to-cycle fluctuations: at 1 sun, the three consecutive cycles gave 0.518, 0.506, and 0.525 kg·m^–2^·h^–1^, and at 3.5 suns 1.683, 1.699, and 1.649 kg·m^–2^·h^–1^, corresponding to maximum deviations of only 2.3 and 2.0%, respectively. Also, there is no salt crust blocking the active coated area within the tested time window (Figure S22 in Supporting Information). The adoption of a floating ISSG configuration, with the surface of the PT film always covered by a thin liquid film, prevents another critical drawback of concentrated brines, that is salt accumulation/crystallization at the evaporating interface, which can partially block pores, reduce the effective wet area, and introduce an additional mass-transfer resistance, further suppressing the evaporation kinetics under otherwise identical PT heating.^69,70^

Finally, to evaluate the relative performance improvement induced by PT NP incorporation, we define the enhancement factor (EF) as the ratio of the NP–CA evaporation efficiency to that of the pure CA membrane and compare the EF of this work with other solar water evaporation systems reported in literature in which the cellulose substrate was used as a blank baseline. While our NP–CA membrane shows an EF of 6.5 under 3.5 sun illumination and 6.2 under 1 sun illumination, literature typically reports EFs around 2 (see Figure 7G and Table S3 in Supporting Information), with a maximum of 5.39,^78^ highlighting the pronounced effect of Fe–Mn–B d NPs in our system.

#### 2.6.2 Solar Water Purification Tests with Fe–Mn–B NP–CA Nanofiber Membranes

Beyond boosting the absolute water evaporation rate, a key requirement for practical deployment is the ability of the Fe–Mn–B based evaporator to deliver high-quality potable water starting from contaminated feeds. We therefore assessed the PT-driven purification performance of the Fe–Mn–B NP-CA nanofiber membrane using three representative classes of pollutants: (i) organic dyes as nonvolatile molecular pollutants typical of textile wastewater, (ii) concentrated NaCl brines (5–10 wt %) as ionic, high-osmotic-strength feeds relevant to desalination, and (iii) trace perfluorooctanoic acid (PFOA) as a persistent, low-volatility, carcinogenic contaminant that challenges many conventional adsorptive or oxidative treatments. The fundamental separation principle is the same in all cases, as ISSG is a phase-change distillation process in which nonvolatile solutes are retained in the liquid feed, while only water vapor traverses the PT interface and is subsequently condensed. In all cases, the NP–CA nanofiber interfacial evaporator was operated in the sealed distillation cell described in Figure 7A, and the condensed vapor was collected and analyzed as the purified product (Figure 7H,I).

Model dye solutions were first prepared with Malachite Green (MG) and rhodamine B (RhB). Under identical simulated solar illumination, the feed solutions displayed the characteristic intense absorption bands of MG and RhB in the visible region, accompanied by vivid green and pink coloration, respectively (Figure 7H,I). After the solar evaporation–condensation cycle, the collected condensates became visually transparent (insets in Figure 7H,I), and their UV–vis spectra showed complete disappearance of the dye absorption peaks, with the residual baseline approaching that of Milli-Q water. We next evaluated desalination performance using NaCl solutions with concentrations of 5 and 10 wt %, representative of saline and brine streams more concentrated than natural seawater (∼3.5 wt %). As shown in Figure 7J, the electrical conductivity of both feed solutions was drastically reduced after solar evaporation, approaching the low conductivity of Milli-Q water. Importantly, the high desalination efficiency is maintained even at 10 wt % NaCl, indicating that the nanofiber membrane and Fe–Mn–B coating tolerate elevated osmotic pressures without performance degradation. Ion leaching from the laser-synthesized Fe–Mn–B nanoparticles in condensed water was verified through ICP–MS analysis, indicating that the levels of Fe, Mn and B in the collected water ensure safety for potable use (see Table S4 in Supporting Information).

Finally, the ability of the system to remove trace, low-volatility contaminants was probed using PFOA as a model of per- and polyfluoroalkyl substances (PFAS). Feed solutions containing PFOA at 1.0 × 10^–6^ and 1.0 × 10^–5^ mol·L^–1^ were subjected to solar-driven distillation, and the PFOA concentration in the condensate was quantified through LC/ESI-MS (Figure 7K). In both cases, PFOA was no more detectable after the treatment (limit of detection, LOD, 9 × 10^–10^ mol·L^–1^), reaching the same level as in PFOA-free Milli-Q water. The suppression of PFOA in the distillate underscores the advantage of a purely phase-change-based purification mechanism, which is not limited by the chemical stability of the solute. Taken together, these results demonstrate that the optimized Fe–Mn–B NP-CA nanofiber evaporator combines high-rate solar steam generation with broad-spectrum water purification capability. The device simultaneously removes colored organic dyes, inorganic salts, and small-molecule PFAS pollutants under purely solar input. This combination of high flux, robustness to salinity, and effective contaminant rejection highlights the potential of multielement Fe–Mn–B PT nanostructures as scalable platforms for solar-driven water treatment.

### 2.7 Life-Cycle Assessment

#### 2.7.1 Life-Cycle Assessment of Fe–Mn–B NP–CA Nanofiber Evaporators

Motivated by the positive PT performance of the heterostructured NPs, the prospective life-cycle implications of an ISSG device based on LAL-derived nanomaterials were studied. The LCA (Figure 8A) highlights the dominant role of NP synthesis conditions in controlling the global warming potential (GWP) of the Fe–Mn–B NP-CA nanofiber evaporators. To isolate the effect of LAL productivity (and the associated electricity intensity) on GWP, we kept constant all nonelectricity foreground inputs (polymer/substrate masses, solvent system, water demand, and downstream fabrication settings), considering three scenarios by varying only the NP supply module. This controlled-variable design ensures that the differences in GWP can be attributed primarily to improvements in NP productivity rather than to changes in the membrane recipe.

**Figure 8:**
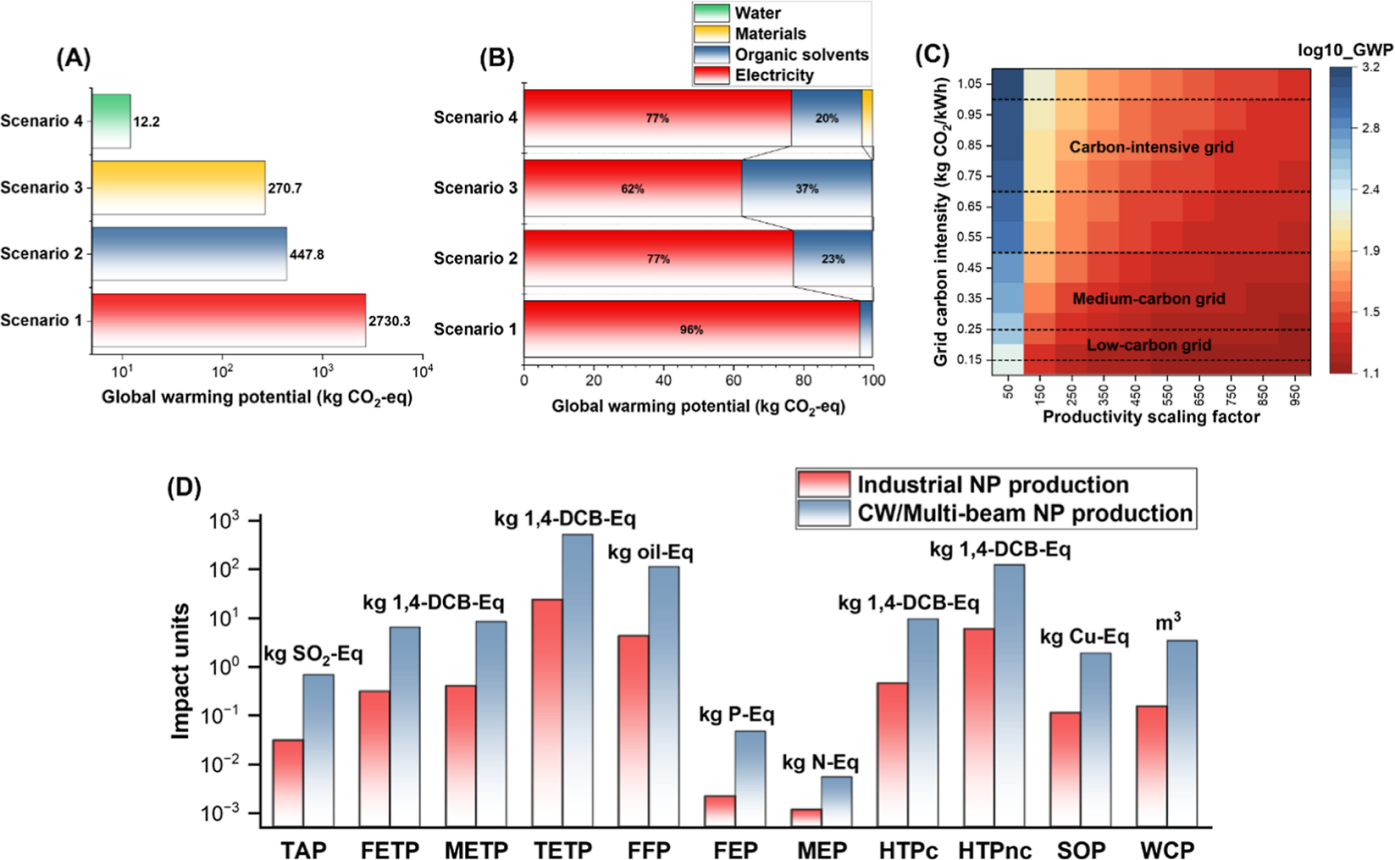
Life-cycle climate performance and impact breakdown of Fe–Mn–B NP-CA nanofiber evaporators under different NP supply scenarios. (A) GWP, kg CO_2_-eq per kg of dried NP-CA nanofiber evaporator for four NP production scenarios: scenario 1 (lab LAL), scenario 2 (optimized LAL), scenario 3 (advanced multibeam LAL), and scenario 4 (industrial NP production). All scenarios assume identical electrospinning and membrane fabrication conditions. (B) Contribution analysis of GWP for each scenario, resolved by major foreground flows (electricity, acetone, DMAc, metal feedstock, cellulose and deionized water); only contributions larger than 3% are shown for clarity. The progressive decrease in the electricity share and the emergence of solvent and metal-feedstock hotspots along scenarios 1–4 illustrate how increased LAL productivity and the use of industrial NP supply shift the dominant climate drivers. (C) Sensitivity heatmap of the prospective climate impact as a function of LAL productivity and electricity carbon intensity. The color scale reports log 10­(GWP) (kg CO_2_-eq per kg evaporator), calculated by scaling the electricity demand with a productivity factor (relative to the baseline laboratory throughput) and varying the grid carbon intensity over a representative range. Horizontal dashed lines indicate illustrative regimes for low-carbon, medium-carbon, and carbon-intensive electricity mixes. (D) ReCiPe 2016 midpoint impact scores for the industrial NP scenario, reported in their native units (e.g., kg SO_2_-eq for TAP, kg 1,4-DCB-eq for FETP, kg oil-eq for FFP, m^3^ for WCP).

For the lab-scale LAL route (scenario 1, lab LAL), the GWP approaches the order of 10^3^ kg CO_2_-eq per kilogram of evaporator membrane, reflecting the low mass productivity of the benchtop high-energy laser setup and the associated electricity demand of the laser system, auxiliary equipment, and postprocessing instrumentation (Figure 8A). In scenario 1, the electricity of the whole chain is effectively spread over a small NP mass. In energy terms, this scenario requires on the order of 10^4^ MJ of electricity per kg of nanofiber, which translates into a GWP of approximately 2.73 × 10^3^ kg CO_2_-eq·kg^–1^ for the evaporator. This magnitude is consistent with previous analyses of lab-scale LAL, which already pointed out that classical LAL configurations can be highly energy-intensive when operated at low yields.^35–38,40^ As the LAL process is progressively optimized, the per-kilogram burden decreases almost proportionally to the gain in NP productivity.

Scenario 2 (optimized LAL) represents a more efficient nanosecond laser system aligned with reports of gram-per-hour productivity using high-repetition-rate ps lasers and fast scanning strategies.^79,80^ Compared with scenario 1, the electricity demand per kilogram of Fe–Mn–B NPs-CA nanofiber is reduced by roughly 1 order of magnitude, and the GWP drops accordingly into the 10^2^–10^3^ kg CO_2_-eq·kg^–1^ range (Figure 8A).

Furthermore, scenario 3 (advanced multibeam LAL) conceptually corresponds to a multibeam industrial laser system, in line with recent demonstrations of multi-kW, multibeam LAL reactors achieving tens to hundreds of grams of colloids per hour.^40,41,80^ Here, the specific electricity use per kilogram of nanofiber is lowered by another factor of 5–10 relative to scenario 2. In our model, this reduces the GWP to the order of 10^2^ kg CO_2_-eq·kg^–1^ range (Figure 8A). Finally, scenario 4 (industrial NP production) represents the long-term ideal limit where Fe–Mn–B NPs are no longer produced by lab-scale LAL, but by a dedicated industrial inorganic NP process with an energy intensity comparable to existing iron-oxide powder production.^81,82^ In practice, we approximate this by replacing the lab-LAL foreground process with an industrial transition metal NP production data set in the background LCA model.^81,82^ For the industrial downstream film-manufacturing stage in scenario 4, we assumed an electricity demand of 22.5 kWh per kg of final nanofiber film, which is consistent with industrial-scale electrospinning LCA inventories reporting ∼30 kWh·kg^–1^ when including solvent recovery and auxiliary unit operations, and we selected a slightly lower value to represent a more process-integrated, continuous production line.^83,84^ In this prospective case the specific electricity demand drops to the order of 10^2^ MJ per kg of nanofiber, and the GWP falls to 12.2 kg CO_2_-eq·kg^–1^ (Figure 8A), i.e. almost 3 orders of magnitude lower than the current lab-LAL practice (scenario 1).

The stacked contributions in Figure 8B resolve how the dominant GWP drivers evolve across these four scenarios. In scenario 1, electricity clearly overwhelms all other flows, accounting for about 96% of the total GWP, with solvents, polymer and water each contributing well below the 1–3% level. This confirms that, at current lab productivity, LAL is essentially an electricity-to-NP converter where almost all climate impact is concentrated in the power used to run the laser, computer, stirring, ultrasonication and centrifugation steps. In scenario 2, the assumed increase in NP productivity and moderate optimization of the synthesis workflow substantially reduce the absolute electricity demand, but electricity still contributes around 77% of the GWP; acetone, mainly from electrospinning preparation and membrane fabrication, emerges as a secondary hotspot (23%). In scenario 3, further improvements in beam utilization (e.g., multibeam operation) shift the balance where electricity drops to 62% of the GWP, while acetone rises to 37%, reflecting that once the laser becomes more energy-efficient, the solvent life cycle begins to matter nearly as much as the power input. In the industrial NP scenario (scenario 4), the contribution profile changes qualitatively and becomes shared between downstream electricity and solvent supply chains. Electricity required for membrane manufacturing remains the dominant driver, contributing 76% of the total GWP (9.27 of 12.18 kg CO_2_-eq·kg^–1^), whereas the organic solvents become the second hotspot (DMAc 12% and acetone 8%). In contrast, the embedded footprint of the upstream metal precursor proxy (magnetite, used here to represent industrial Fe/Mn-oxide nanopowders supplying the NP mass fraction) contributes only ∼1.5%. Thus, once NP supply is approximated by an industrial powder data set, the climate impact is no longer governed by the low productivity of lab-scale LAL, but by the electricity and solvent intensity of the film fabrication workflow.

The scenario comparison demonstrates a hotspot transition, in which electricity dominates impacts at laboratory throughput, while nonelectric contributions (e.g., solvent supply/management and downstream processing) become increasingly influential as productivity improves. Figure 8C makes this dependency explicit by mapping the prospective log 10­(GWP) as a function of productivity scaling and grid carbon intensity. Two practical implications follow: (i) productivity improvements deliver the largest climate benefit under carbon-intensive electricity mixes, where the electricity term dominates the footprint and reductions in unit electricity demand translate directly into large GWP decreases; (ii) as productivity increases and the electricity supply is progressively decarbonized, the marginal benefit of further gains in either lever diminishes, and the relative importance of nonelectric contributions increases, particularly solvent supply/management and downstream manufacturing.

#### 2.7.2 Beyond GWP: Multicategory LCA Comparison of NP Production Routes

To move beyond climate change, Figure 8D reports the absolute ReCiPe 2016 midpoint scores for the industrial NP scenario across multiple impact categories, using their native units (e.g., kg SO_2_-eq for terrestrial acidification potential (TAP), kg 1,4-DCB-eq for freshwater ecotoxicity potential (FETP), marine ecotoxicity potential (METP), terrestrial ecotoxicity potential (TETP), carcinogenic—human toxicity potential (HTPc) and noncarcinogenic—human toxicity potential (HTPnc), kg oil-eq for fossil resource potential (FFP), kg P-eq for freshwater eutrophication potential (FEP), kg N-eq for marine eutrophication potential (MEP), kg Cu-eq for surplus ore potential (SOP), m^3^ for water consumption potential (WCP)). As expected, global warming and fossil resource scarcity are both substantial, reflecting the remaining dependence on grid electricity and fossil-based chemical production. The highest scores are observed for human carcinogenic toxicity and freshwater ecotoxicity, which are driven primarily by upstream production of organic solvents (acetone, DMAc) and specialty chemicals in the electrospinning dope, as well as by metal mining and processing. By contrast, categories such as land use and water consumption remain comparatively small for the nanofiber production, indicating that the evaporator fabrication step itself is not land- or water-intensive. This multicategory profile shows that decarbonising power supply alone is insufficient, while toxicity-related indicators become the next bottleneck once electricity is cleaned up. Overall, Figure 8A–D maps a plausible transition pathway for LAL-based NP synthesis from today’s energy-intensive laboratory practice toward future low-impact industrial manufacturing. The comparison between Scenarios 1–3 demonstrates order-of-magnitude gains in LAL productivity through higher average laser power, multibeam architectures, improved optical coupling to the target and reduced idle time, which translates almost linearly into reductions in GWP. Scenario 4 indicates that, if NP supply can approach the energy intensity of existing transition metal NP powder production while maintaining the compositional flexibility of LAL, the overall climate footprint of Fe–Mn–B NP–CA nanofiber evaporators becomes comparable to that of other porous membranes reported in the literature.^81^ Once industrial NP production is assumed, the remaining hotspots lie in polymer/solvent manufacture and electricity mix rather than in the NP synthesis itself. At that stage, further improvements should target green solvent management (closed-loop acetone/DMAc recovery, replacement by water-based or bioderived solvents), lower-toxicity polymers, and renewable electricity sourcing (e.g., PV-powered LAL and electrospinning lines), which would directly reduce the toxicity and resource-depletion hotspots highlighted in Figure 8D.

From a broader cross-disciplinary perspective, these results establish design rules for LAL-derived NP that extend well beyond solar evaporation. First, LAL processes should be evaluated and optimized not only for NP quality but also for energy-normalized productivity (mass removed per kWh), as this metric directly governs GWP and fossil resource use. Second, when LAL is integrated with downstream shaping steps such as electrospinning, cryogelation, coating, or printing, the choice of solvent system and its recovery strategy becomes a dominant factor for toxicity-related impact categories, comparable in importance to the laser process itself. Third, coupling high-throughput LAL reactors with renewable or low-carbon electricity has the potential to transform this contamination-free synthesis route into a genuinely green platform for producing nanostructured coatings and membranes. At the same time, the toxicity-related hotspots in Figure 8D point toward solvent substitution and solvent-recovery technologies as the next Frontier in making LAL-enabled nanofiber evaporators a genuinely sustainable option for solar water evaporation and purification.

### 2.8 Conclusions

In conclusion, we establish and validate a DOE-guided LAL strategy to discover earth-abundant Fe–Mn–B multielement NPs for interfacial solar steam generation. From an experimental data set of 24 LAL syntheses spanning laser pulse duration, solvent composition, and target stoichiometry, a prediction model was trained on three application-relevant metrics (PT peak temperature, solar-spectrum-weighted absorption, and a productivity-linked concentration indicator). Inverse model guidance, refined by literature-informed conditions, led to the adoption of a Fe_10_Mn_24_B_66_ target and high-energy LAL in acetone to produce a compositionally heterogeneous Fe/Mn/B@C nanoarchitecture. When integrated onto CA substrates, the optimized Fe–Mn–B NP–CA membrane delivers an evaporation rate of 2.402 ± 0.05 kg·m^–2^·h^–1^ under illumination at 3.5 sun, exceeding the broadband absorbing Au NP reference (1.725 ± 0.07 kg·m^–2^·h^–1^) under identical conditions. Translation from a commercial CA membrane to an electrospun CA nanofiber membrane preserves the area-normalized rate (2.40 ± 0.05 vs 2.40 ± 0.10 kg·m^–2^·h^–1^ at 3.5 sun) within the presently tested membrane formats and increases total vapor production by enlarging the active interfacial area, while achieving an enhancement factor of 6.5 under 3.5 sun illumination and 6.2 under 1 sun illumination relative to blank CA baselines (typical literature value is 2). The resulting evaporators sustain desalination up to 10 wt % NaCl and enable broad-spectrum purification, including dyes and the PFAS model contaminant PFOA. Finally, scenario-based LCA is used to quantify prospective manufacturing implications and to identify actionable levers. The analysis indicates electricity as the dominant hotspot at laboratory throughput, with solvent supply and downstream processing becoming increasingly important as productivity improves. Together, these results position LAL as a chemistry-minimized route to PT nanoheterostructures that retain performance upon proof-of-concept 5× active area scale-up in the presently tested membrane formats, and outline throughput, power sourcing, and solvent management as the primary engineering constraints for translating laboratory performance toward lower-impact water treatment technologies.

## 3 Methods

### 3.1 Fe–B and Mn–B NP Synthesis

In the LAL synthesis configuration, four different Fe–B and Mn–B targets with atomic percent of 1:2 and 2:1 were submerged in 40 mL of either pure ethanol (100%), a 1:1 ethanol-to-water mixture, or pure water (100%), as shown in Figure 2A. Solid ablation targets were achieved from homogeneous powder mixture (all from Sigma-Aldrich) of pure metals including iron (Fe, 99.5 at %, 7 ± 1.25 μm) and manganese (Mn, 99.8 at %, 45 ± 15 μm) and boron (B, 99.8 at %, 1 ± 0.5 μm) using a Carver hydraulic pellet press set at 517 MPa for 5 min.

All NP synthesis experiments were conducted under ambient air conditions using a pulsed fiber laser coupled with a Master Oscillator Power Amplifier and a 12 mm spiral scanning pattern made with a galvanometric scanner, all controlled by the EZCAD software. The scanning pattern allowed for uniform ablation over the target area at 5 m/s. The pulse duration was set as 20 and 200 ns, the laser power was set as 100% for all experiments (11.4 and 11.8 W for, respectively, 20 and 200 ns pulses), the frequency was set at 50 kHz, and the duration of each synthesis was set at 5 min. After LAL, the optical absorption of all synthesized NP solutions was recorded with UV–visible spectroscopy. Then, the samples were centrifuged to remove the solvent and subsequently washed three times with DI water. During each washing step, the residual ethanol was gradually replaced by water; after centrifugation, the supernatant was discarded, and the sediment was redispersed in fresh water for the next cycle. After the synthesized NP solution was dispersed in water, all of them were set to an equal absorbance of 0.6 at 200 nm in a 2 mm quartz cell. The 24-experiment parameter table is shown as Table S1 in the Supporting Information.

The Fe–Mn–B a and b samples were both obtained from the bulk Fe_10_Mn_24_B_66_ target immersed in 120 mL of either a 1:1 (v/v) ethanol–water mixture or pure acetone, with the fiber laser operated at 0.28 mJ/pulse (100% laser power, 14.2 W), 25 ns, 50 kHz, 5 m/s for 5 min each synthesis. The Fe–Mn–B c and d samples were both obtained from the bulk Fe_10_Mn_24_B_66_ target immersed in 120 mL of either a 1:1 (v/v) ethanol–water mixture or pure acetone. A Q-switched Nd:YAG laser (1064 nm) operated at 50 mJ/pulse (2.5 W), 6 ns, 50 Hz, 10 mm/s, for 90 min each synthesis. The laser beam was focused onto the target surface using an *f* = 100 mm lens, while the target was scanned in a circular Archimedean spiral pattern with a maximum diameter of 10 mm. Each complete scan cycle was finished within 200 s by mounting the ablation cell on a motorized XY translation stage (Standa), controlled by a two-axis stepper motor, a DC motor controller, and a custom-written LabVIEW program. Argon bubbling was applied during LAL to suppress flammability risks.

#### 3.1.1 Fabrication of Gel-Embedded NPs

Agarose-embedded NPs were prepared as schematized in Figure 3A,B. Agarose (molecular biology grade; Fisher Scientific; cat. no. 10766834) stock solution was prepared by dissolving 400 mg of agarose powder in 20 mL deionized water using a hot air gun with continuous stirring until the solution became fully transparent (∼90–95 °C). The molten agarose was kept liquid and stirred gently to prevent premature gelation and deaerated by vacuum pumping before use. For blank gels, 2.0 mL deionized water was mixed with 1.0 mL molten agarose in a 1 cm disposable polystyrene cuvette for fluorescence spectroscopy. For NP gels, 2.0 mL calibrated NP dispersion was combined with 1.0 mL molten agarose in the same cuvette type and sonicated for 30 s to ensure uniform distribution, avoiding overheating. Samples were cooled to room temperature (∼25 °C) for 5–10 min and stored sealed with cuvette caps and parafilm until PT testing.

#### 3.1.2 Fabrication of NP–CA Membrane

A hydrophilic cellulose acetate filter (0.45 μm cutoff, 25 mm filter diameter, VWR International) was mounted on a syringe and loaded with the freshly sonicated NP dispersion, with the volume adjusted such that the absorbance at 200 nm was normalized across all samples, which was subsequently filtered through the membrane. To enhance NP adhesion, the filter was rinsed with 1 mL of an aqueous solution containing 1% tetraethyl orthosilicate (TEOS, Sigma-Aldrich) and filtered again. Residual liquid was removed by pushing air through the syringe, and the filter in a breaker was predried at 70 °C for 3 h on a hot plate. Finally, the filter was cut to the desired size and left to dry under ambient conditions for an additional 24 h before use.

#### 3.1.3 Fabrication of NP–CA Electrospun Membrane

Electrospinning was selected as the fabrication technique due to its ability to produce continuous polymer fibers with nanoscale diameters and interconnected porous networks. Cellulose acetate (acetyl content ≈39–40 wt %, average molecular weight ≈30 000 g·mol^–1^, Sigma-Aldrich) was used as received. Acetone (ACS reagent grade, ≥99.5%) and *N*,*N*-dimethylacetamide (DMAc, anhydrous, ≥99%) were purchased from Sigma-Aldrich (Merck KGaA, Darmstadt, Germany) and used without further purification. In a typical preparation, a CA precursor solution (15 wt %) was obtained by dissolving 1.5 g of CA in 8.5 g of a binary solvent mixture of acetone and DMAc (2:1 w/w). The mixture was magnetically stirred at 45 °C until a homogeneous, bubble-free solution was obtained.

Electrospinning was carried out using a conventional electrospinning setup consisting of a syringe pump (Harvard 11 Pico Plus, Harvard Apparatus, USA), a DC high-voltage power supply (Gamma High Voltage Research, Ormond Beach, FL, USA) and a grounded flat aluminum-foil collector, enclosed in a PMMA chamber to minimize air drafts and stabilize the ambient conditions. The CA solution was loaded into a 5 mL plastic syringe fitted with a 12.5 mm blunt-tip stainless-steel needle and fed at 1.2 mL·h^–1^. A positive voltage of 18 kV was applied to the needle, while the collector was grounded; the tip-to-collector distance was fixed at 20 cm. All electrospinning experiments were performed under ambient laboratory conditions (21.9 °C, relative humidity ≈84.5%), enabling the stable formation of a Taylor cone and the deposition of uniform nanofiber mats onto filter paper supports. After electrospinning, the membranes were dried at room temperature to remove residual solvent before further use.

To functionalize the nanofibers with NPs, a post-treatment deposition strategy was employed. The dried nanofiber film was mounted on a gooch crucible with fine filtration support and prewetted with deionized water. Subsequently, 16 mL of NP dispersion (1.44 mg/mL) was applied in 1 mL increments under gentle vacuum suction, ensuring uniform infiltration and adsorption of NPs throughout the fiber network. Finally, 500 μL of TEOS was drop-cast onto the fiber membrane to reinforce NP adhesion and improve the mechanical integrity of the composite structure. Through this approach, electrospun nanofiber membranes embedded with a polystyrene support ring (outer diameter: 60 mm; inner diameter: 35 mm) were fabricated, enabling controlled NP loading and improved operational stability compared with the previously used NP-coated commercial CA films.

### 3.2 Characterization

UV–visible absorption spectroscopy was performed with JASCO V770 UV–vis–NIR spectrometer in 2 mm quartz cells. Powder XRD analysis was performed with a Panalytical X’Pert Powder diffractometer equipped with a Cu tube (40 kV, 40 mA), a BBHD mirror, a spinner, and a PIXcel detector. The samples were deposited on Si zero-background substrates by drop-casting and drying at room temperature. The diffraction patterns were processed using MDI JADE 9 software (Materials Data Inc., Livermore, CA) with the COD database for phase identification. Whole-pattern fitting (WPF) Rietveld refinement was carried out within JADE 9. The background was modeled and subtracted using the automatic background-fitting routine implemented in the software, and the Bragg peak profiles were described by pseudo-Voigt functions. The quality of the refinements was evaluated using the weighted profile *R*-factor (*R*_wp_), the expected *R*-factor (*R*_exp_), and the goodness-of-fit parameter *G* = *R*_wp_/*R*_exp_ as reported by JADE 9.

Scanning electron microscopy (SEM) and energy-dispersive X-ray spectroscopy (EDS) were carried out using a Zeiss Sigma HD field-emission scanning electron microscope (Carl Zeiss, Germany) equipped with a Schottky FEG source, a backscattered-electron detector, and two secondary-electron detectors (InLens and Everhart–Thornley). The instrument was coupled with an Oxford Instruments EDS detector operated in energy-dispersive mode for X-ray microanalysis. Measurements were performed at an accelerating voltage of 20 kV.

HRTEM, STEM-SE, STEM-BSE, HAADF-STEM and STEM-EDS analysis were performed with a Cold-FEG S/TEM JEOL JEM F200 operated at 200 kV. Elemental analysis mapping was performed using a JEOL 100 mm^2^ silicon drift energy EDX spectrometer. Samples were drop casted at room temperature on copper grids coated with a carbon film.

XPS spectra were acquired in an ultrahigh vacuum (UHV) chamber operating at a working pressure below 5 × 10^–8^ mbar. Samples were drop-cast at room temperature on Au-coated Cu substrates. The measurements were performed with Al Kα radiation (*h*ν = 1486.7 eV), with pass energies of 20 eV for B and C and 50 eV for Mn and Fe. Sputtering was performed at 1.5 kV for 5 min with Ar^+^ ions. The substrate Au 4f binding energy (84 eV) was used to calibrate the spectra and the XPS peak 4.1 software for their analysis.

The presence of residual traces of Fe, Mn and B in the purified water samples, and in the reference untreated Milli-Q water, was assessed with ICP–MS using an Agilent Technologies 7700x ICP–MS (Agilent Technologies International Japan, Ltd., Tokyo, Japan). The instrument was equipped with an octupole collision cell operating in kinetic energy discrimination mode for the removal of polyatomic interferences and argon-based interferences. Samples digestion was performed with nitric acid (Carlo Erba, 65%) at 100 °C for 1 h.

Static water contact angles were measured using an LSA 50 surface analyzer (LSA 50, LAUDA Scientific GmbH, Lauda-Königshofen, Germany) operated in sessile-drop mode at room temperature (25 °C) under ambient laboratory humidity. Deionized water droplets of 50 μL were gently deposited onto the sample surface in manual dispensing mode. Immediately after deposition, a sequence of 120 images was recorded at intervals of 0.5 s, and the contact angle of each frame was determined by digital image analysis using the circle-fitting algorithm implemented in the SurfaceMeter software supplied with the instrument. For each sample, the quasi-static contact angle was obtained by averaging the values over the time interval where the drop profile was stable, and the reported values correspond to the mean ± standard deviation of at least three droplets deposited at different positions on the surface.

The conductivity of the saline feed solutions (5 and 10 wt % NaCl) before and after desalination was measured with a benchtop conductivity meter (edge HI2030, Hanna Instruments, Italy) equipped with a four-ring platinum conductivity probe (HI763100) and automatic temperature compensation. The instrument was calibrated daily using standard KCl solutions (1.3004 mS·cm^–1^ at 21 °C) according to the manufacturer’s procedure. For each sample, conductivity values were recorded after thermal equilibration at 21.0 ± 0.5 °C, and the reported values represent the average of three independent measurements.

The concentration of PFOA in the feed solutions and in the condensed water collected after ISSG was determined by liquid chromatography-electrospray ionization mass spectrometry (LC/ESI-MS). Samples were analyzed on a Thermo Scientific Dionex Ultimate 3000 HPLC system coupled to a Thermo Scientific LTQ XL linear ion-trap mass spectrometer (Thermo Fisher Scientific) equipped with an ESI source. Chromatographic separation was performed on an InfinityLab Poroshell 120 EC-C18 column (2.1 × 100 mm, 2.7 μm) using water containing 5 mM ammonium acetate (eluent A) and methanol (eluent b) in a gradient of increasing b (from 20 to 60% in 5 min, to 100% in 16 min, isocratic at 100% for 2 min) at a flow rate of 0.30 mL·min^–1^; the injection volume was 10 μL. The ESI source was operated in negative-ion mode, with a spray of 2.5 kV and a source temperature of 250 °C. Optimized values for auxiliary gas flows were the following: sheath gas = 35 au, auxiliary gas = 10 au, sweep gas = 0 au PFOA was quantified in selected reaction monitoring (SRM) by monitoring the transition from the deprotonated ion, [M – H]^−^ at *m*/*z* 413, to the fragment [M – H – CO_2_]^−^ at *m*/*z* 369. Perfluorononanoic acid (PFNA) was used as the internal standard monitoring the transition from [M – H]^−^ at *m*/*z* 463 to the fragment [M – H – CO_2_]^−^ at *m*/*z* 419, in SRM, and the calibration curve was built considering the ratio of the signals of PFOA and PFNA. The limit of detection (LOD) for PFOA was 9 × 10^–10^ M, and the limit of quantification (LOQ) was 3.9 × 10^–9^ M.

### 3.3 PT Experiments

A tungsten-halogen lamp was used for PT experiments of 24 Fe–B and Mn–B NP embedded gels. The emission profile of the lamp was registered with an Avantes spectroradiometer (AvaSpec-ULS3648 USB2-UA-FC/PC) calibrated for absolute irradiance measurements from 200 to 1100 nm and equipped with a fiber detector with a cosine diffusor. The portion of the emission spectrum from 1100 to 2500 nm was obtained by fitting the 200–1100 curve with the Plank’s law. A Fresnel lens with a focal length of 99 mm was used to focus the lamp light onto the sample. For the measurement with the liquid solution, the samples were placed at the focal distance corresponding to a spot size of 12.4 ± 0.5 mm and width of 8.9 ± 0.5 mm. A thermal camera model FLIR E5 was used to capture the calibrated digital thermographic infrared images of the heated samples.

Solar steam generation experiments were carried out in a beaker containing 200 mL of deionized water, using a floating device supporting either the NP-coated cellulose membrane or the NP-coated electrospun CA membrane. The floating device consisted of a holed circular polystyrene foam ring and a snap-fit Teflon holder used to fasten the substrate. For the NP-coated cellulose membrane, the polystyrene ring had an external diameter of 40 mm and an internal diameter of 14 mm, whereas for the NP-coated electrospun CA membrane, the ring dimensions were 60 mm (outer diameter) and 35 mm (inner diameter). The beaker was irradiated with an AM 1.5G solar simulator (LOT-Quantum Design, AM 1.5G) equipped with a normal-incidence accessory, positioned at 5 cm for 3.5 sun intensity and 23.4 cm for 1 sun intensity from the nanofiber surface, under ambient laboratory conditions. The liquid mass loss for both the bare cellulose substrate and the NP-coated evaporation membranes was measured in triplicate using a KERN PLE-N digital balance over 60 min. The local surface temperature of the evaporator was monitored with a FLIR E5 infrared thermocamera. The evaporation efficiency (η) of the ISSG was calculated using the following formula^10,85^

4 $$\eta =\frac{m_{net}h_{LV}}{C_{opt}q_{sol}}$$

where *q*_sol_ is the nominal solar irradiance at 1 sun, and *C*_opt_ is the corresponding optical concentration factor. The net evaporation rate per unit area *m*_net_ is calculated after subtracting dark evaporation. *h*_LV_ denotes the total phase-change enthalpy, comprising both sensible heat and the latent heat of vaporization. The values used for the efficiency calculation and results are summarized in Table S2 of the Supporting Information.

### 3.4 DOE Regression Analysis

A DOE was conducted with 24 experimental runs (Table S1) varying atomic target composition (Fe_2_B, FeB_2_, Mn_2_B, MnB_2_), solvent (water, water/ethanol 1:1, ethanol), and pulse duration. Three responses were quantified: (i) convolution area, defined as the overlap integral between the *A*_200_ = 0.6-normalized UV–vis absorbance and the solar spectral irradiance over 200–2500 nm; (ii) a concentration indicator, calculated as the NP solution volume (mL) after normalizing the absorbance at 200 nm to 0.6 in a 2 mm quartz cuvette, multiplied by the visible-range convolution area (380–780 nm), thereby reflecting LAL productivity weighted by optically useful solar absorption; and (iii) the peak temperature reached by each NP sample under broadband visible-NIR irradiation. The 380–780 nm range was selected to exclude UV-only absorption features with negligible solar overlap and to avoid NIR regions affected by water absorption. UV–vis spectra were acquired once per run; three selected NP samples in the initial experiment data set were remeasured with two scans, giving CV (coefficient of variation) ≤ 0.741% in integrated absorbance area (coefficient of variation ≤0.741%), which confirms the negligible impact of the instrumental measurement error. Temperature was recorded twice per run and averaged with standard deviations around 0.3 °C. The normalized dilution volume used in the concentration indicator was determined by adjusting each dispersion to a fixed absorbance at 200 nm; therefore, its uncertainty is directly propagated from the absorbance repeatability and was considered negligible. Regression modeling was performed in JMP Pro using candidate full-factorial and second-order (quadratic) effects; terms were selected by stepwise regression with BIC as the stopping rule (eqs 1–3). Finally, the fitted response surfaces were used for inverse prediction via Derringer–Suich desirability-based multiresponse optimization,^48,49^ where individual desirability functions were defined for each response and combined into an overall desirability to simultaneously maximize the three performance indicators and identify an optimal synthesis window. To estimate pure error and quantify batch-to-batch reproducibility at the optimum, independent replicate syntheses showed highly consistent UV–vis responses after *A*_200_ calibration, the integrated absorbance area exhibited a CV of 1.28% in the 380–780 nm region and 5.25% over 200–2500 nm, while the peak temperature showed a within-sample variation of ∼0.8 °C.

### 3.5 Life Cycle Assessment

A cradle-to-gate LCA was conducted to quantify the environmental burdens associated with the production of Fe–Mn–B NP-loaded CA nanofiber membranes. The primary goal was to identify the dominant contributors to GWP and to evaluate how improvements in LAL productivity could translate into lower climate impacts along a plausible scale-up pathway (scenarios 1–4). The assessment was performed in openLCA v2.5.0 using the ecoinvent v3.9.1 (cutoff, UPR) database^82^ as the background system, and impact assessment was carried out with ReCiPe 2016 v1.0.3 Midpoint (H). The functional unit (FU) was defined as 1 kg of final NP-nanofiber evaporator membrane at the factory gate (no use-phase and no end-of-life modeling). The foreground system includes: (i) NP synthesis (when explicitly modeled), (ii) polymer solution preparation and electrospinning, and (iii) NP loading/filtration and auxiliary downstream handling. Background processes were taken from ecoinvent (materials, electricity supply, and upstream chemical production). Packaging, capital goods, infrastructure, and laboratory building overheads were excluded unless otherwise stated.

To isolate the role of LAL productivity (and thus electricity intensity of NP supply) on GWP, a controlled-variable design was adopted for scenarios 1–3: all nonelectricity foreground inputs were kept constant (polymer/substrate masses, solvent system, water demand, and downstream fabrication settings), while only the NP supply module was modified.

Solvent use was modeled on a net-consumption basis to reflect closed-loop recovery and reuse at scale. Specifically, acetone (and other volatile solvents where applicable) was entered into the life cycle inventory as the makeup solvent required per FU after recovery, rather than the gross solvent circulation. In practice, recovered solvent condensed during concentration/drying steps (e.g., rotavapor condensation) was assumed to be reused in subsequent batches, and only the unrecovered fraction contributed to virgin solvent demand

5 $$m_{net}=m_{gross}\left(1-\eta_{rec}\right)$$

where η_rec_ is the assumed recovery efficiency.

To construct the sensitivity heatmap in Figure 8C, we report the total climate impact (GWP, kg CO_2_-eq per kg evaporator) as a function of productivity scaling and grid carbon intensity. The total GWP was written as the sum of (i) an electricity-dependent term and (ii) a fixed nonelectric background term taken from the scenario-based life-cycle inventory

6 $${GWP}_{total}\left(x,CI_{grid}\right)={GWP}_{non‐elec}+{GWP}_{elec}\left(x,CI_{grid}\right)$$

The electricity contribution was expressed as

7 $${GWP}_{elec}\left(x,CI_{grid}\right)=E\left(x\right)\cdot CI_{grid}$$

where CI_grid_ (kg CO_2_/kWh) was scanned over a representative range to emulate different electricity mixes, and *E*(*x*) denotes the electricity demand per functional unit. The productivity scaling factor *x* was defined relative to the baseline laboratory throughput, and *E*(*x*) was scaled accordingly (i.e., higher productivity corresponds to lower unit electricity intensity under otherwise identical processing assumptions). Importantly, all nonelectric contributions (e.g., solvent supply/management, materials, and downstream processing) were kept constant for a given inventory; therefore, the variation across the heatmap arises only from the electricity term *E*(*x*)·CI_grid_, while GWP_nonelec_ acts as an additive baseline. The resulting values were visualized as log 10­(GWP_total) to accommodate the wide dynamic range and improve interpretability.

## Supplementary Material



## Notes

Supporting Information 24-experiment parameter table for Fe–B and Mn–B NP synthesis (Table S1); heating–cooling curves and pre/postirradiation absorbance spectra of Fe_2_B 1–6 NP gels (Figures S1 and S2); heating–cooling curves and pre/postirradiation absorbance spectra of FeB_2_ 1–6 NP gels (Figures S3 and S4); heating–cooling curves and pre/postirradiation absorbance spectra of Mn_2_B 1–6 NP gels (Figures S5 and S6); heating–cooling curves and pre/postirradiation absorbance spectra of MnB_2_ 1–6 NP gels (Figures S7 and S8); supplementary DOE interaction plots for the three NP performance metrics (Figure S9); heating–cooling curves of Fe_10_Mn_24_B_66_ NPs a–c (Figure S10); pre/postirradiation absorbance spectra of Fe_10_Mn_24_B_66_ NPs a–d (Figure S11); surface and cross-sectional EDS mapping of Fe–Mn–B NPs d (Figure S12); XPS analysis of Fe–Mn–B d NPs (Figure S13); HRTEM and STEM images of Fe–Mn–B d NPs (Figure S14); HRTEM and STEM images of pristine target powder for the Fe–Mn–B d sample (Figure S15); SEM/EDS/XRD characterization of Fe–Mn–B NPs c (Figure S16); mass loss versus irradiation time of Fe_10_Mn_24_B_66_ NPs d, Fe_10_Mn_24_B_66_ NPs c, Au NPs, and blank CA commercial membranes under 3.5-sun irradiation (Figure S17); time-dependent contact angle of the CA nanofiber membrane before and after NP coating (Figure S18); 1-sun mass loss data of NP-CA and blank CA nanofiber membranes (Figure S19); continuous 3 h mass loss and time-dependent evaporation-rate data of NP-CA nanofiber membranes in 10 wt % NaCl at 1 sun and 3.5 suns (Figures S20 and S21); membrane photographs before and after continuous-operation tests (Figure S22); repeated restart-based cycling data showing cumulative mass loss and time-dependent evaporation rates in 10 wt % NaCl at 1 sun and 3.5 suns (Figures S23 and S24); parameters and calculations for solar-to-vapor efficiency (Table S2); literature comparison of evaporation performance and enhancement factor for cellulose-based solar evaporators (Table S3). ICP–MS analysis of Fe, Mn and B (Table S4) (PDF)
