# Supplementary material for: Optimisation of Laser-Synthesized Heterostructured Multielement Nanoparticles for Solar Steam Generation and Water Purification
Source: ACS Appl Mater Interfaces. 2026 Jun 2;18(23):32859–81. doi: 10.1021/acsami.6c05452 (PMC13288389; doi:10.1021/acsami.6c05452)
Supplement: Supplementary file 1 [file am6c05452_si_001.pdf]

Supporting Information

Optimisation of laser-synthesized heterostructured multielement nanoparticles for solar steam generation and water purification

Runpeng Miao,[1,*] Martina Roso,[2] Linbo Jin,[1] Marco Bortolussi,[1] Ester Marotta,[1] Vincenzo Amendola[1,*]

[1] Department of Chemical Sciences, University of Padova, Via Marzolo 1, 35131 Padova, Italy

[2] Department of Industrial Engineering, University of Padova, Via Marzolo 9, 35131 Padova, Italy

* runpeng.miao@phd.unipd.it; vincenzo.amendola@unipd.it

Table S1. The 24-experiment parameter table for Fe-B and Mn-B NP synthesis. The target compositions listed in the table refer to the nominal atomic ratio formulations of the ablation targets used as DOE input variables.

Target composition (sample #)	Pulse duration (ns)	Solvent	Repetition rate	Scanning speed
Fe_2B (1)	20	Ethanol	50 kHz	5 m/s
Fe_2B (2)	20	H_2O/Ethanol (1:1)		
Fe_2B (3)	20	H_2O		
Fe_2B (4)	200	Ethanol		
Fe_2B (5)	200	H_2O /Ethanol (1:1)		
Fe_2B (6)	200	H_2O		
FeB_2 (1)	20	Ethanol		
FeB_2 (2)	20	H_2O /Ethanol (1:1)		
FeB_2 (3)	20	H_2O		
FeB_2 (4)	200	Ethanol		
FeB_2 (5)	200	H_2O /Ethanol (1:1)		
FeB_2 (6)	200	H_2O		
Mn_2B (1)	20	Ethanol		
Mn_2B (2)	20	H_2O /Ethanol (1:1)		
Mn_2B (3)	20	H_2O		
Mn_2B (4)	200	Ethanol		
Mn_2B (5)	200	H_2O /Ethanol (1:1)		
Mn_2B (6)	200	H_2O		
MnB_2 (1)	20	Ethanol		
MnB_2 (2)	20	H_2O /Ethanol (1:1)		
MnB_2 (3)	20	H_2O		
MnB_2 (4)	200	Ethanol		
MnB_2 (5)	200	H_2O /Ethanol (1:1)		
MnB_2 (6)	200	H_2O		

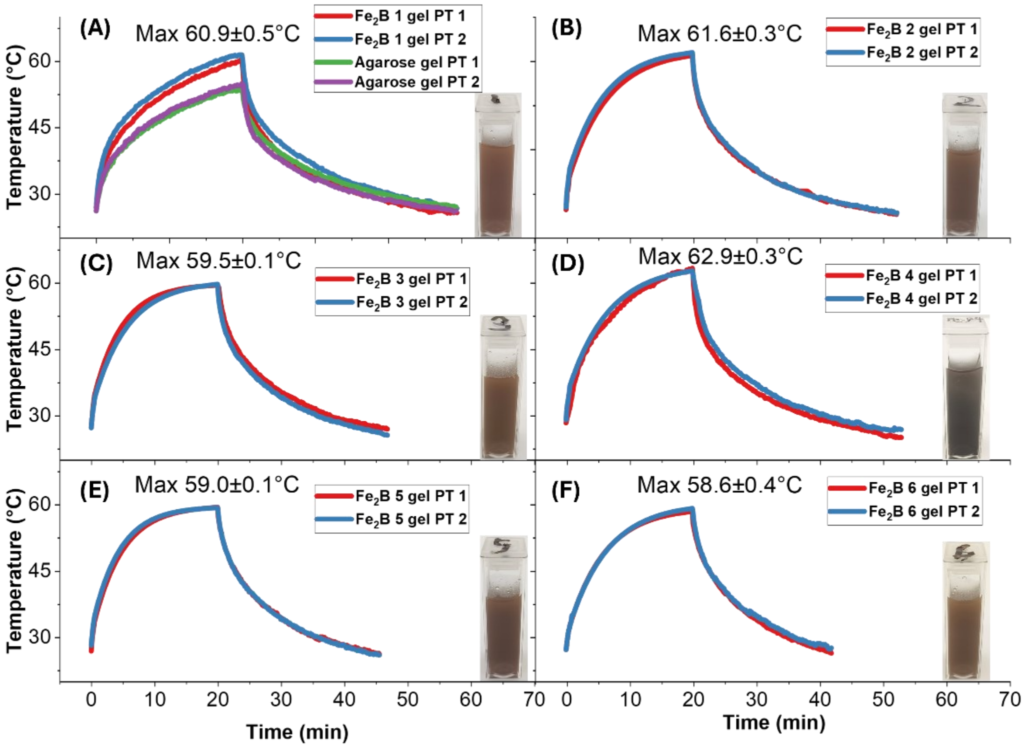

Figure S1. (A-F) The heating-cooling curve of agarose gel blank and Fe_2B 1-6 NP gel. PT 1 and 2 are two repeated experiments.

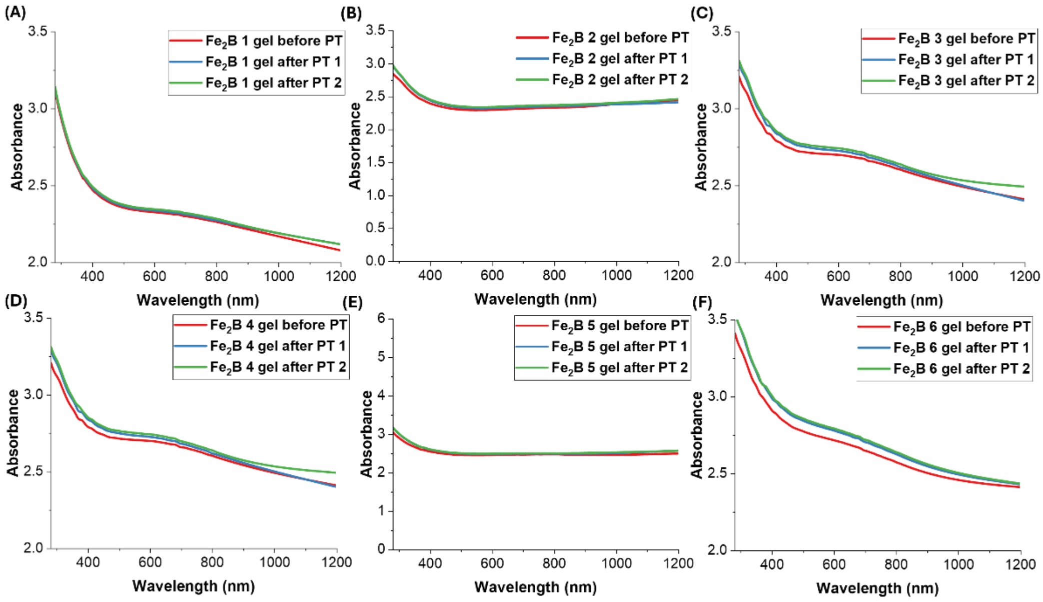

Figure S2. (A-F) The absorbance spectra of Fe_2B 1-6 NP gel before and after the photothermal heating experiment. PT 1 and 2 are two repeated experiments.

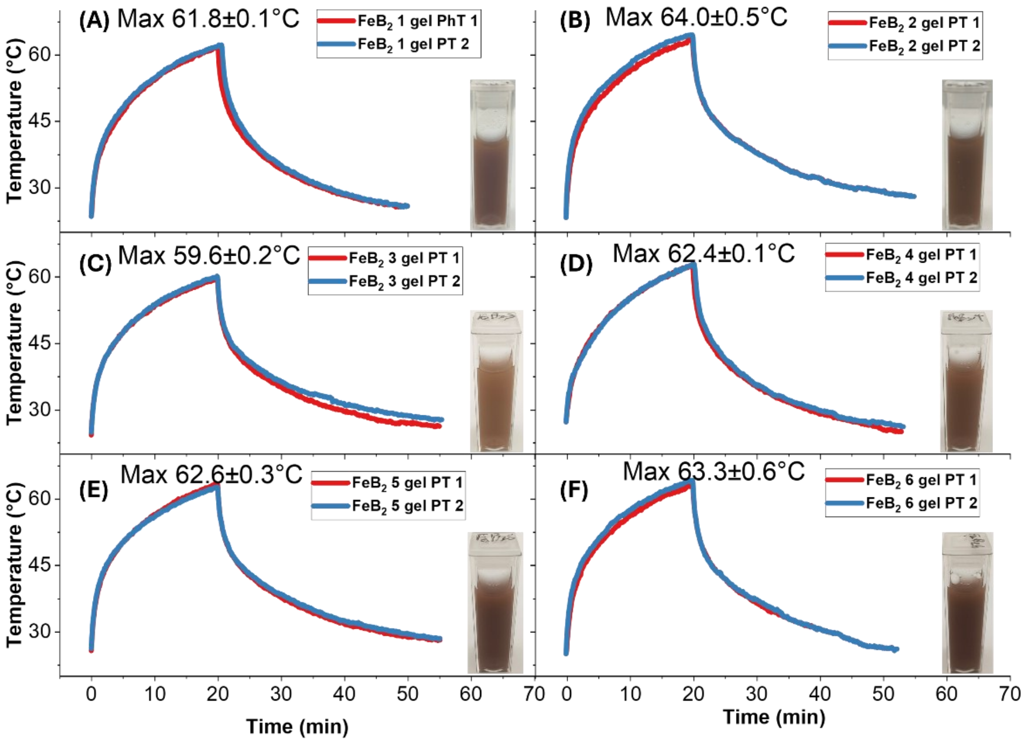

Figure S3. (A-F) The heating-cooling curve of FeB_2 1-6 NP gel. PT 1 and 2 are two repeated experiments.

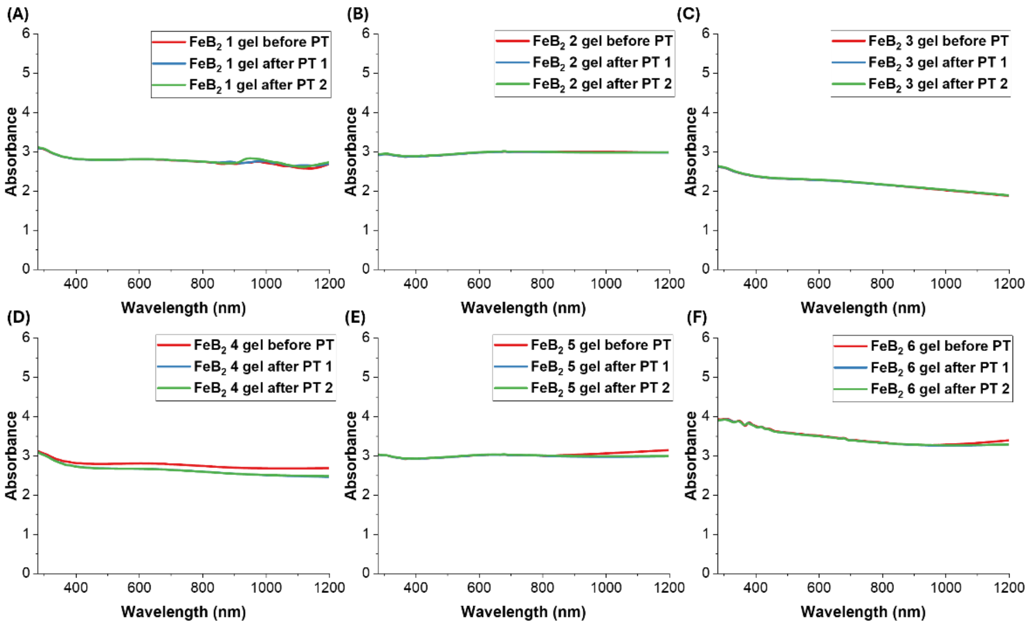

Figure S4. (A-F) The absorbance spectra of FeB_2 1-6 NP gel before and after the photothermal heating experiment. PT 1 and 2 are two repeated experiments.

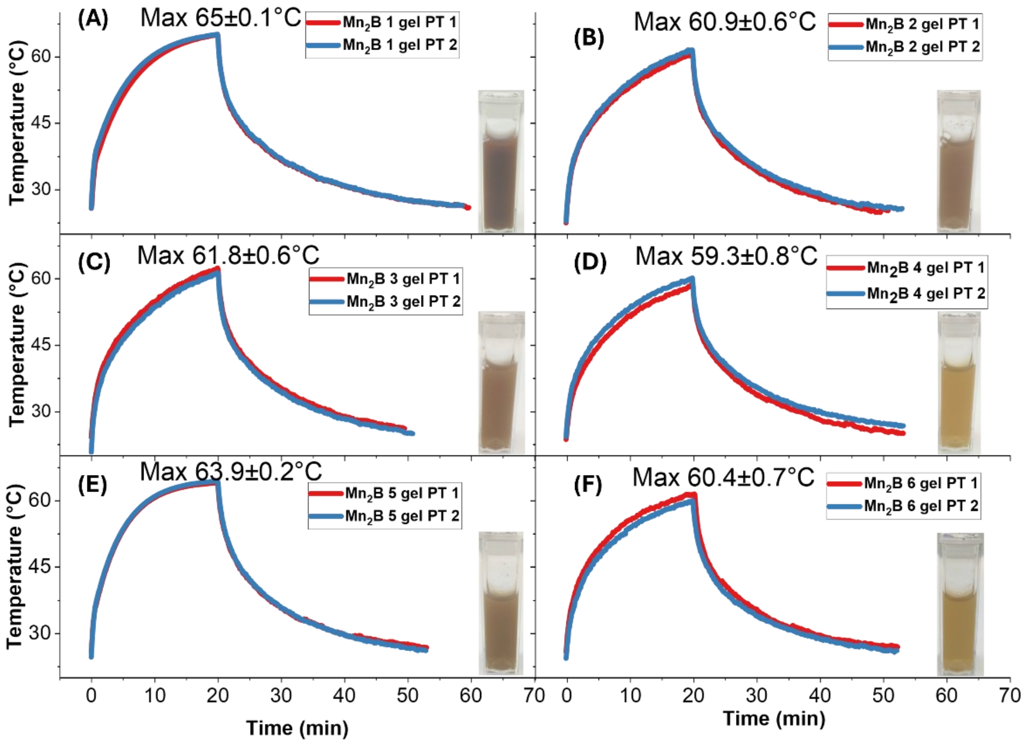

Figure S5. (A-F) The heating-cooling curve of Mn_2B 1-6 NP gel. PT 1 and 2 are two repeated experiments.

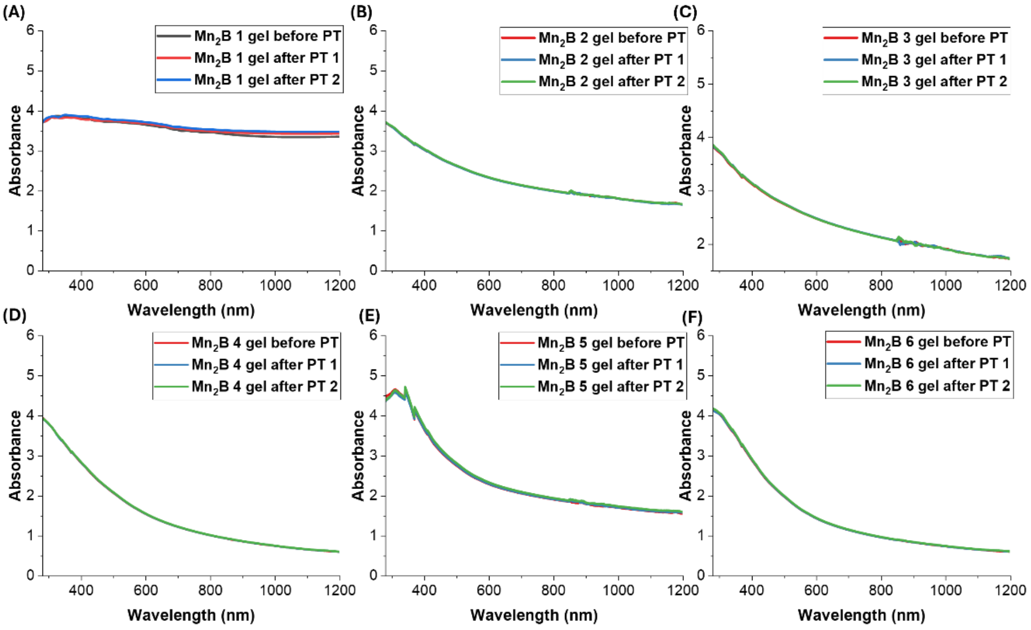

Figure S6. (A-F) The absorbance spectra of Mn_2B 1-6 NP gel before and after the photothermal heating experiment. PT 1 and 2 are two repeated experiments.

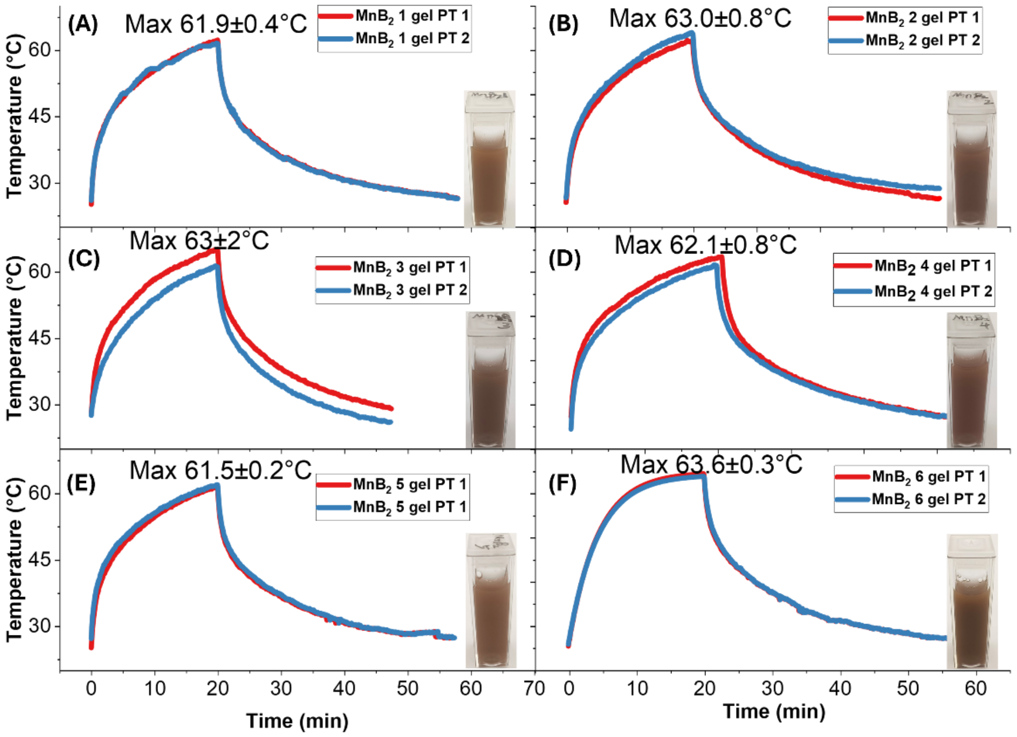

Figure S7. (A-F) The heating-cooling curve of MnB_2 1-6 NP gel. PT 1 and 2 are two repeated experiments.

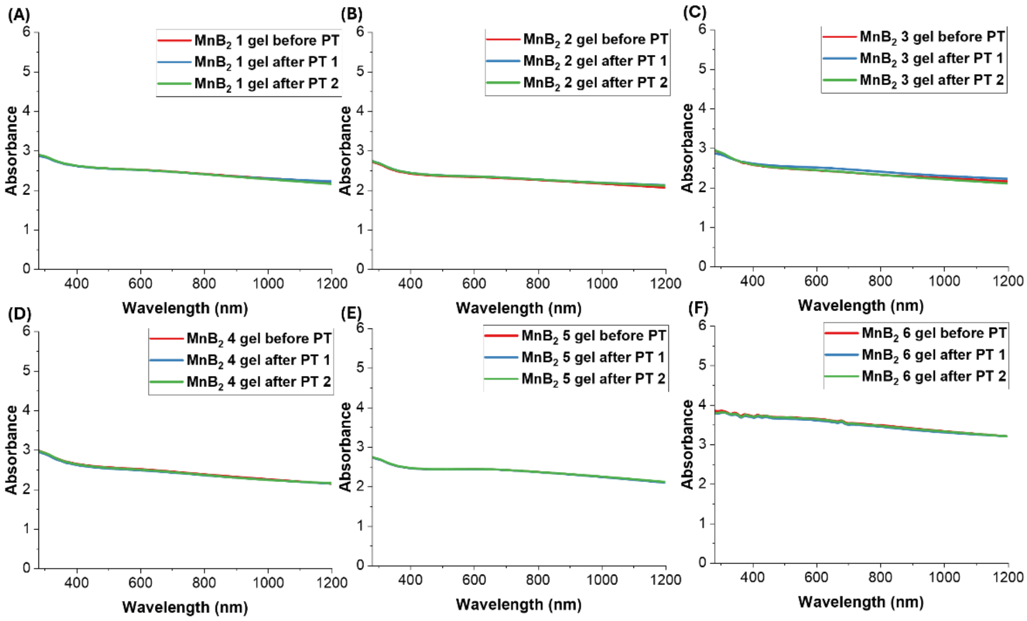

Figure S8. The absorbance spectra of MnB_2 1-6 NP gels (A-F) before and after the photothermal heating experiment. PT 1 and 2 are two repeated experiments.

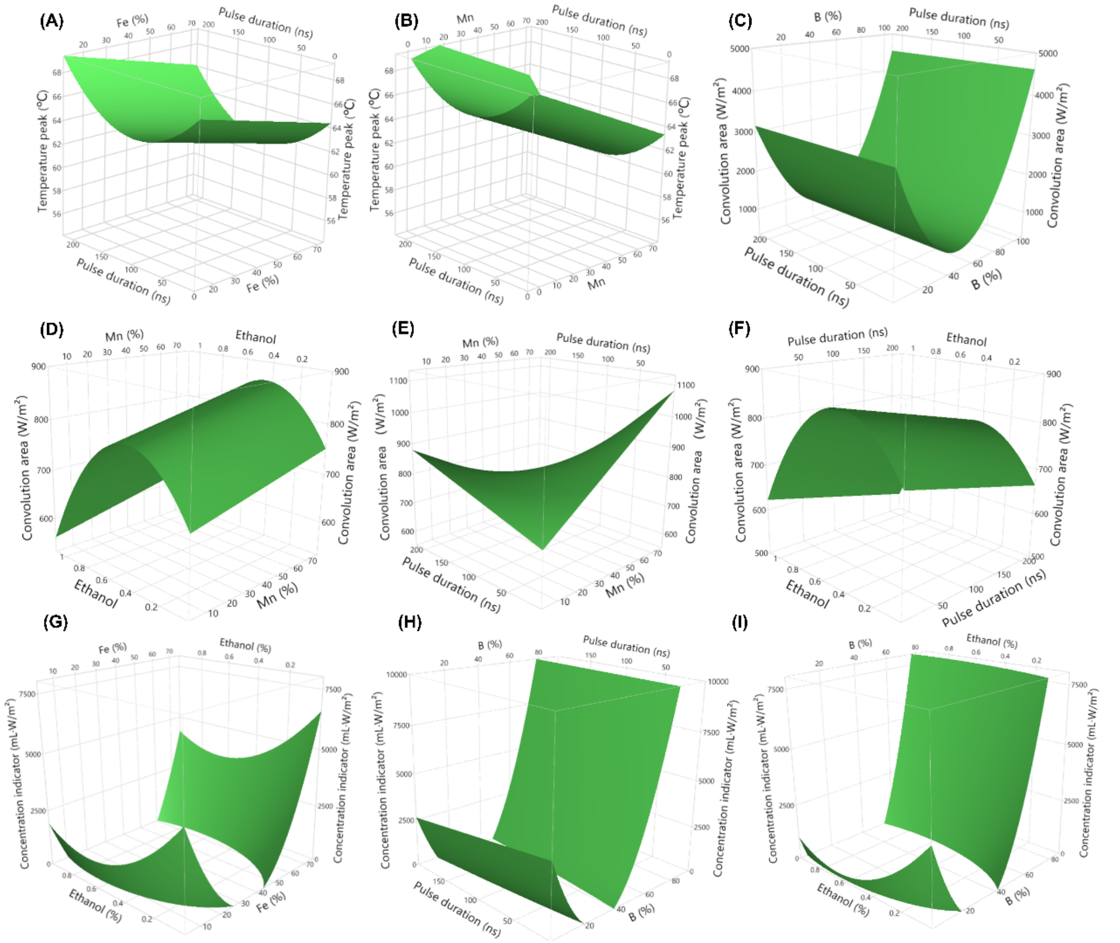

Figure S9. The coupling effect of (A) the at% of Fe in the target and pulse duration; (B) the at% of Mn in the target and pulse duration on the reached temperature peak of synthesized NPs. The coupling effect of (C) the at% of B in target and pulse duration; (D) the at% of Mn and Ethanol in solvent; (E) the at% of Mn in target and pulse duration; (F) pulse duration and the at% of ethanol in solvent on the convolution area between solar irradiation spectrum and the absorbance spectra of synthesized NPs. The coupling effect of (G) the percentage of Fe in target and Ethanol in solvent; (H) the percentage of B in target and pulse duration; (I) the at% of B in target and Ethanol in solvent on the concentration indicator of synthesized NPs.

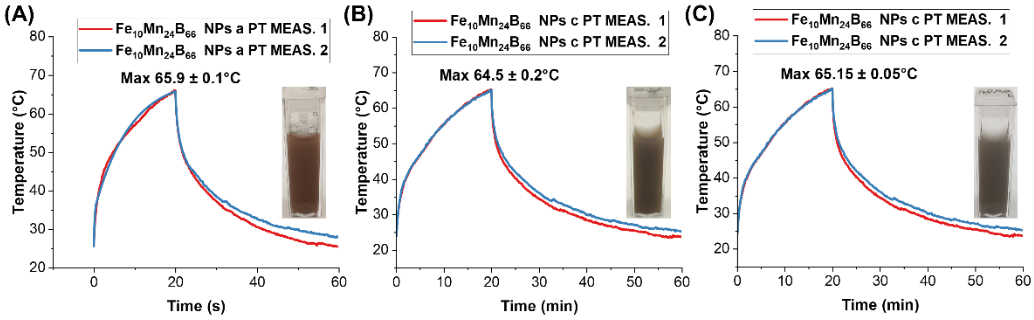

Figure S10. Heating-cooling curves of Fe-Mn-B NPs samples a (A), b (B) and c (C).

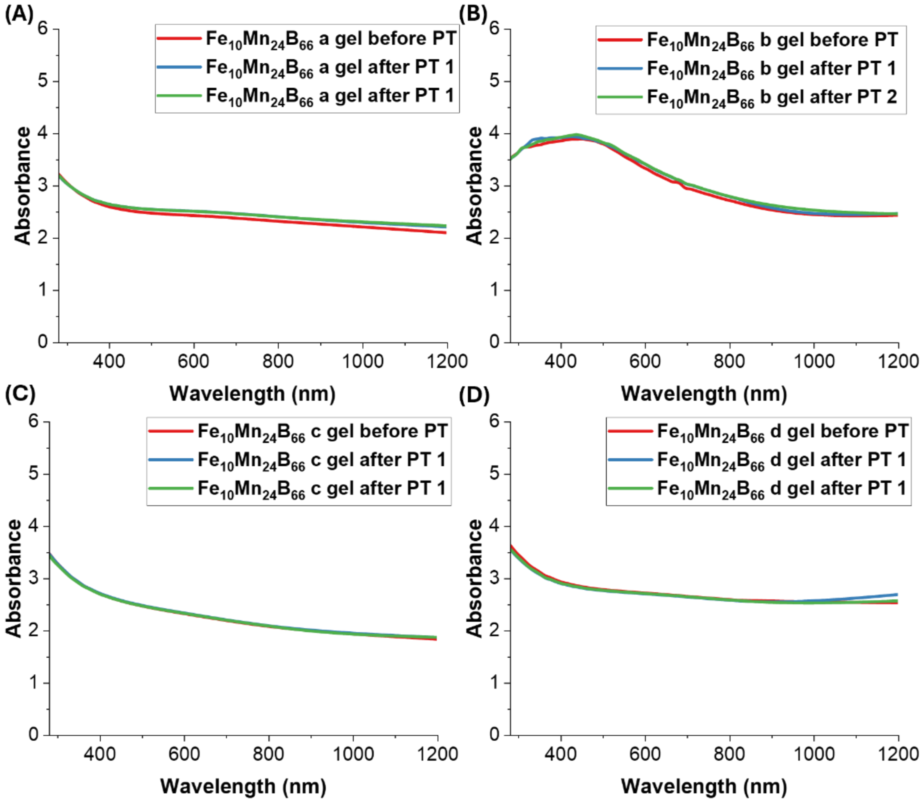

Figure S11. The absorbance spectra before and after the photothermal heating experiment of gels of $Fe_{10}Mn_{24}B_{66}$ NPs a – synthesized in 1:1 ethanol-to-water mixture with 0.28 mJ/pulse, 50 kHz laser pulses (A); b – synthesized in acetone with 0.28 mJ/pulse, 50 kHz laser pulses (B); c – synthesized in 1:1 ethanol-to-water mixture with 50 mJ/pulse, 50 Hz laser pulses (C); d – synthesized in acetone with 50 mJ/pulse, 50 Hz laser pulses (D). PT 1 and 2 are two repeated experiments.

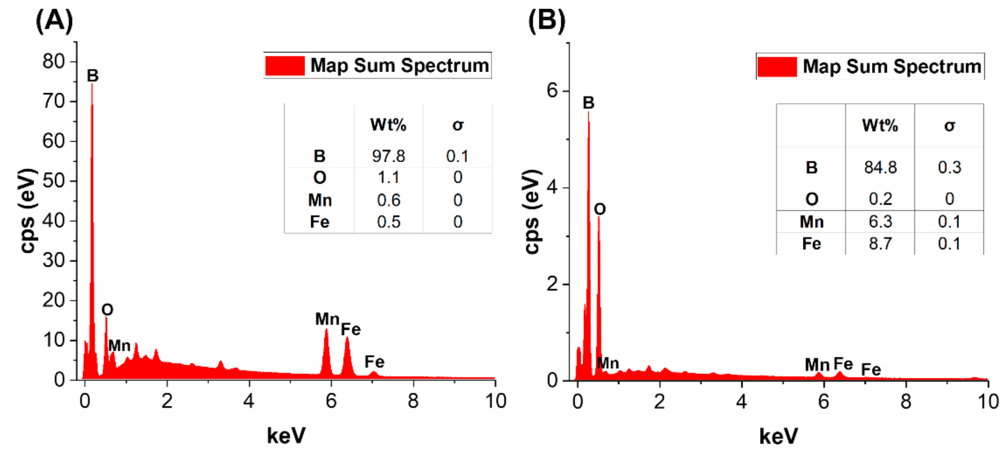

	Wt%	σ
B	97.8	0.1
O	1.1	0
Mn	0.6	0
Fe	0.5	0

	Wt%	σ
B	84.8	0.3
O	0.2	0
Mn	6.3	0.1
Fe	8.7	0.1

Figure S12. (A) Surface element mapping EDS analysis of Fe-Mn-B NPs d. (B) Cross-sectional element mapping EDS analysis of Fe-Mn-B NPs d.

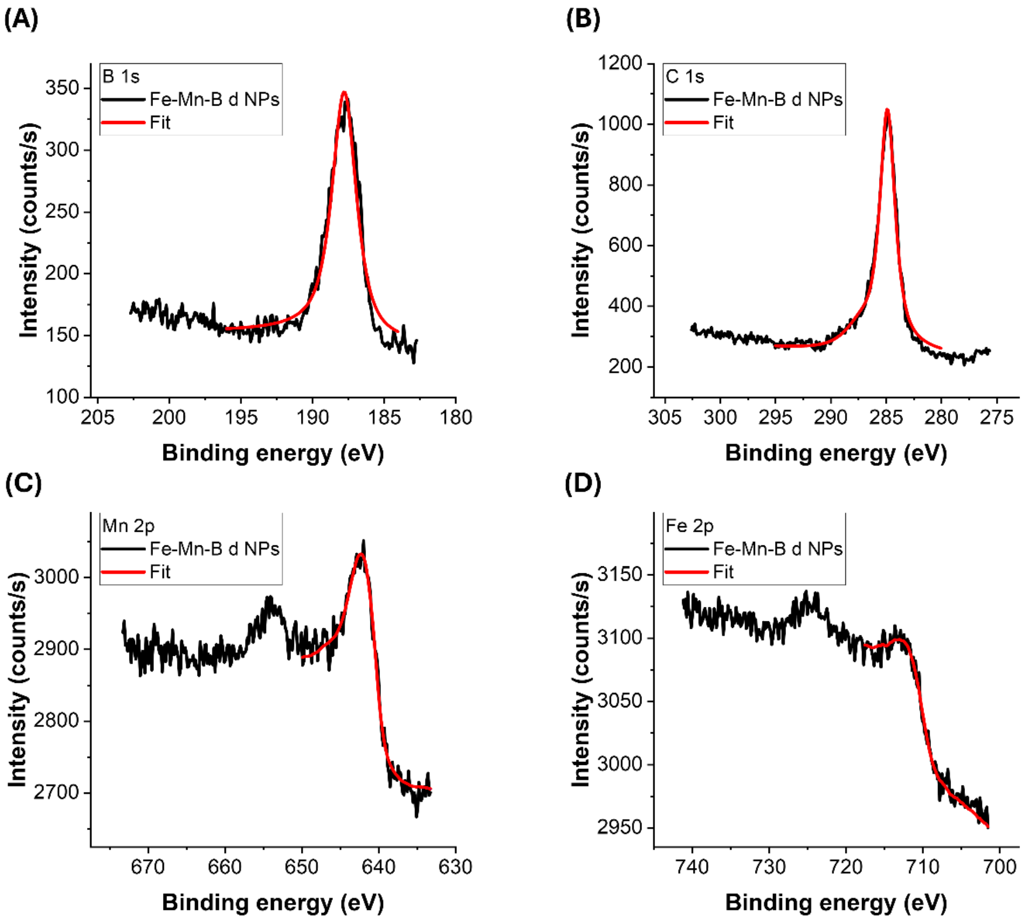

Figure S13. XPS spectra of B 1s (A), C 1s (B), Mn 2p (C) and Fe 2p (D) collected in the Fe-Mn-B d sample.

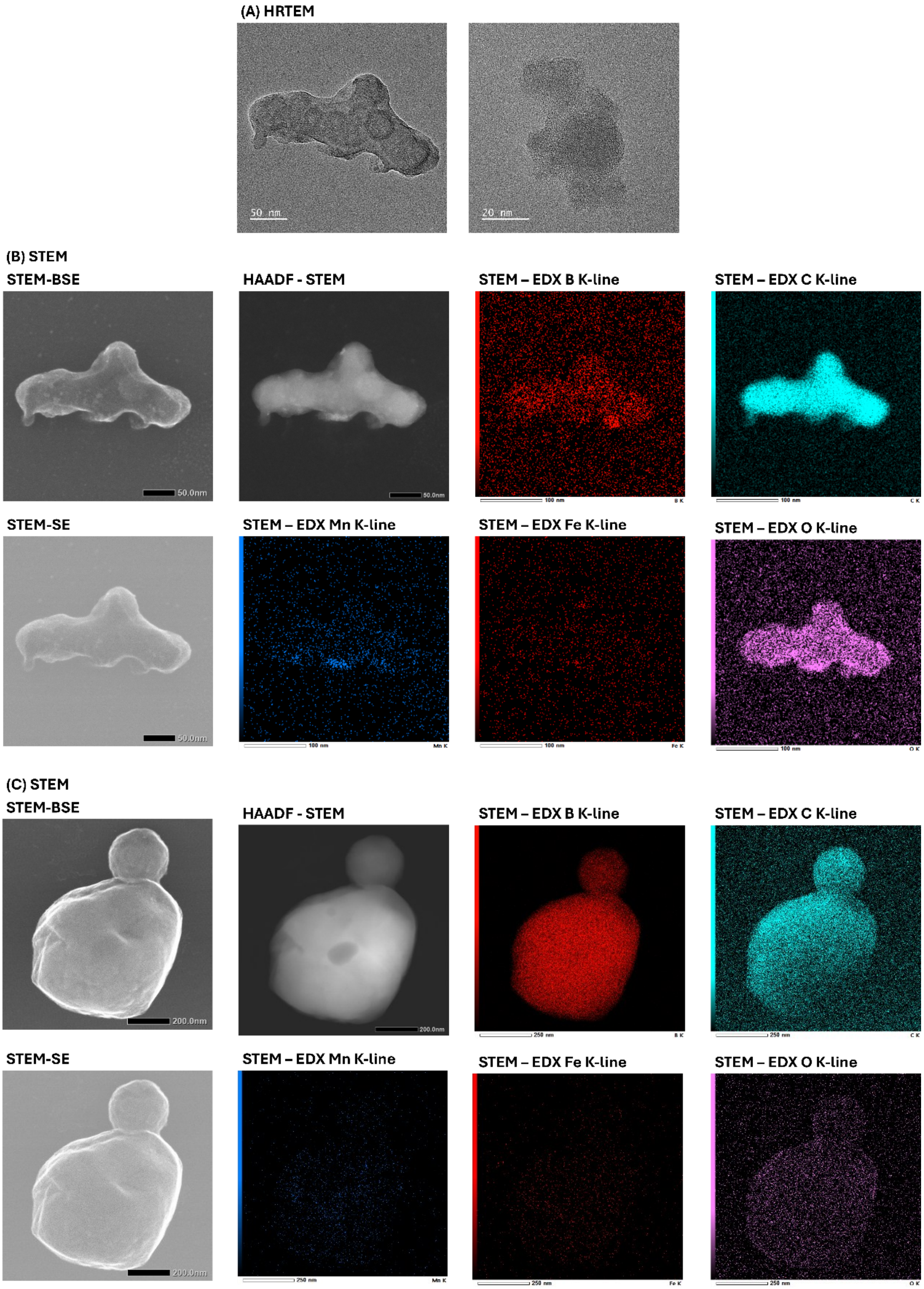

Figure S14. HRTEM (A) and STEM (B-C) analysis of NPs with different size from the Fe-Mn-B d sample.

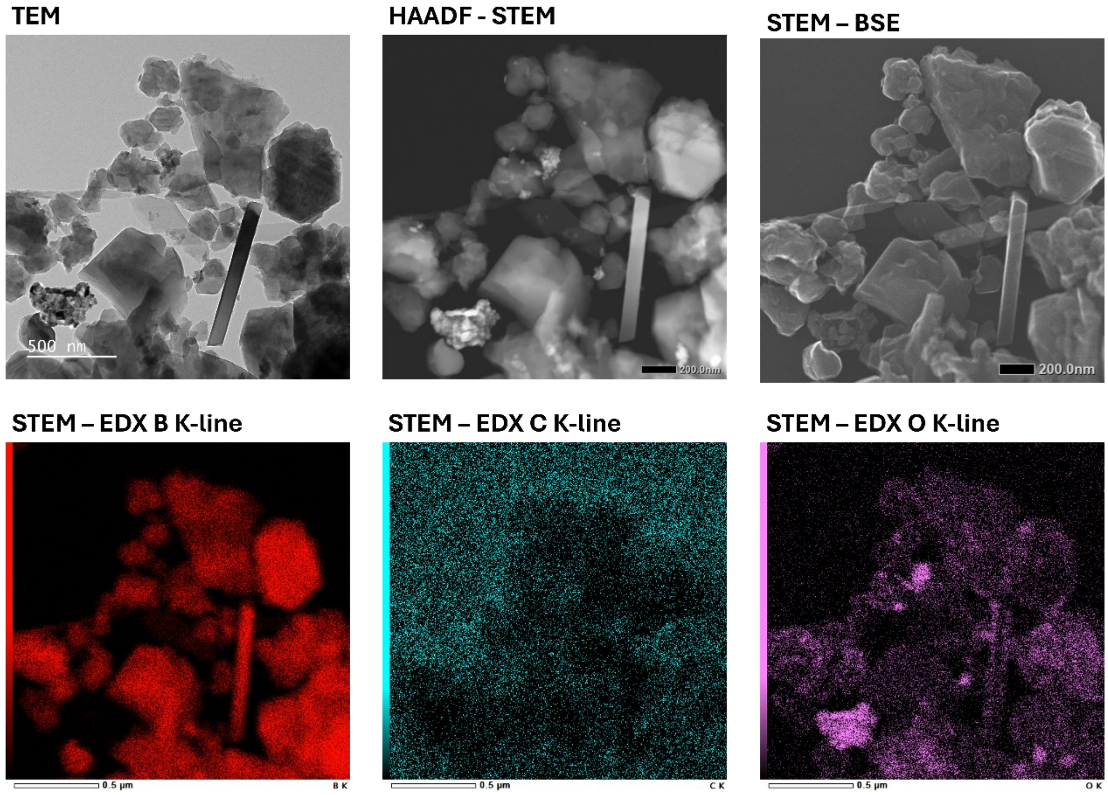

Figure S15. HRTEM and STEM analysis of the pristine powder used for LAL synthesis of Fe-Mn-B d NPs.

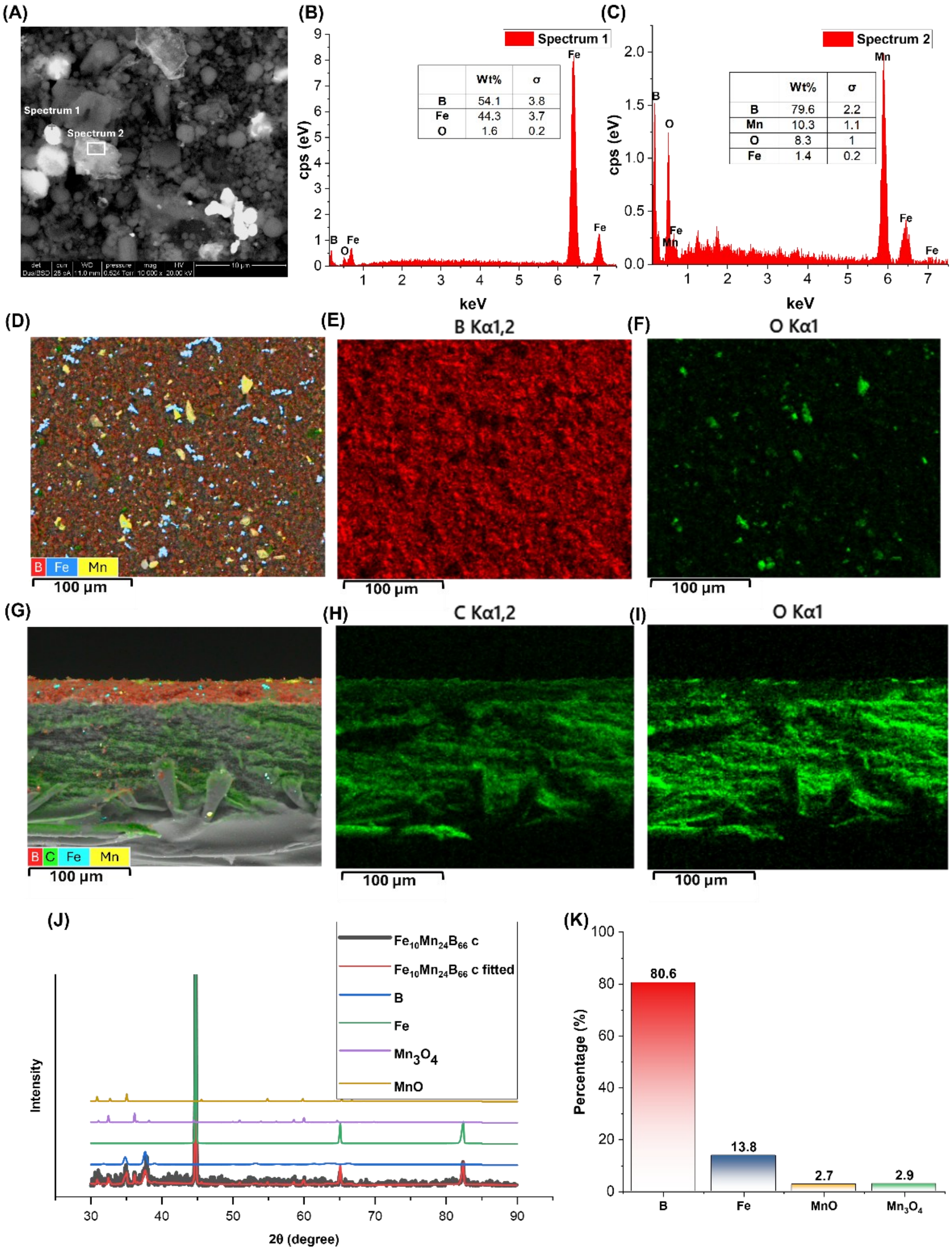

Figure S16 (A) The SEM morphology analysis of Fe-Mn-B NPs c. (B-C) The point element analysis of spectra 1, 2 in the SEM image (A). (D) EDS elemental mapping of Fe-Mn-B c NPs, showing B in red, Fe in blue, and Mn in yellow. (E) Broad and phase-segregated distribution of B across the entire region is shown in (D). (F) O enrichment is preferentially associated with Mn-rich regions, with lower intensity on Fe-rich regions in (D). (G) Cross-sectional SEM morphology of Fe-Mn-B NPs c deposited on a cellulose filter, with elemental mapping of C (green), B (red), Fe (cyan), and Mn (yellow). (H) Broad distribution of C across the Fe-Mn-B region in (G). (I) Scattered distribution of O within the Fe–Mn–B cross-section in (G). (J) The XRD analysis and Rietveld refinement fitting of Fe-Mn-B NPs c. (K) The quantitative phase composition obtained from Rietveld refinement.

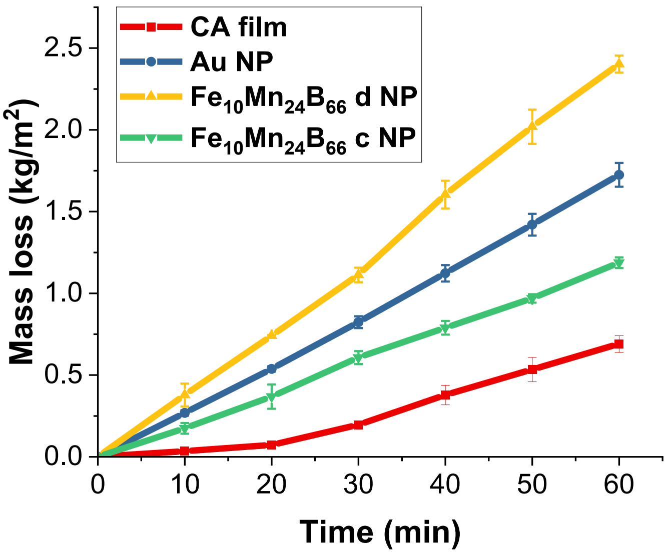

Figure S17. The mass loss versus irradiation time (0-60 min) of $Fe_{10}Mn_{24}B_{66}$ NPs d, $Fe_{10}Mn_{24}B_{66}$ NPs c, Au NPs on CA commercial membranes and the blank CA substrate under sunlight simulator at 3.5-sun intensity.

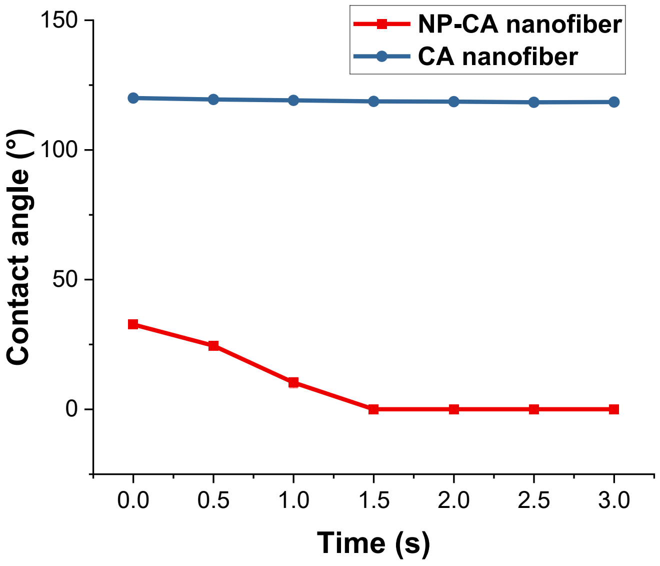

Figure S18. Time-dependent contact angle of a water droplet on the nanofiber surface. The variation of the contact angle was recorded as a function of contact time, illustrating the wettability behavior of the nanofibers.

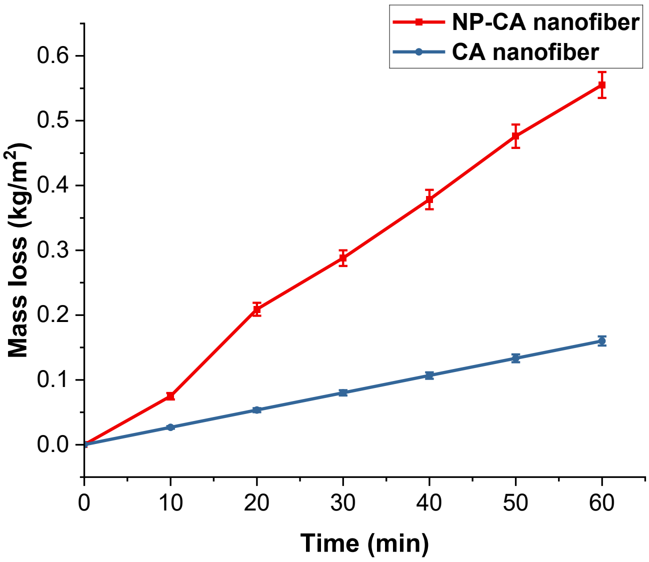

Figure S19. The mass loss of $Fe_{10}Mn_{24}B_{66}$ d NP - CA electrospun nanofiber and the blank CA nanofiber under 1-sun intensity.

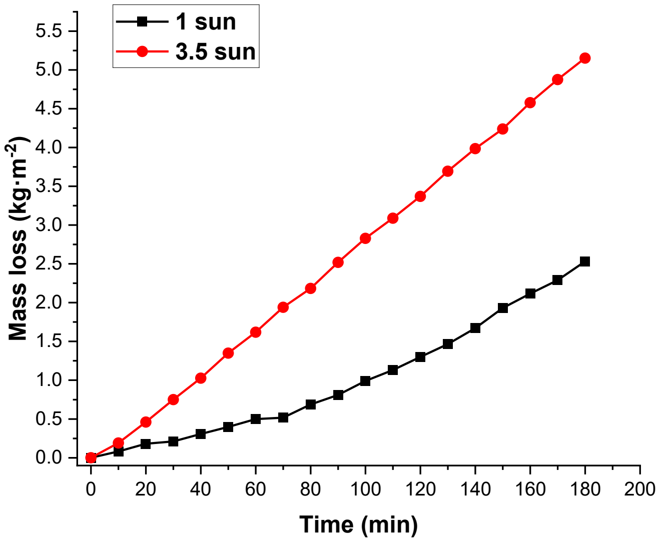

Figure S20. Mass loss versus irradiation time for the NP-CA nanofiber membrane under 1 sun, 10 wt% NaCl, continuous 3 h.

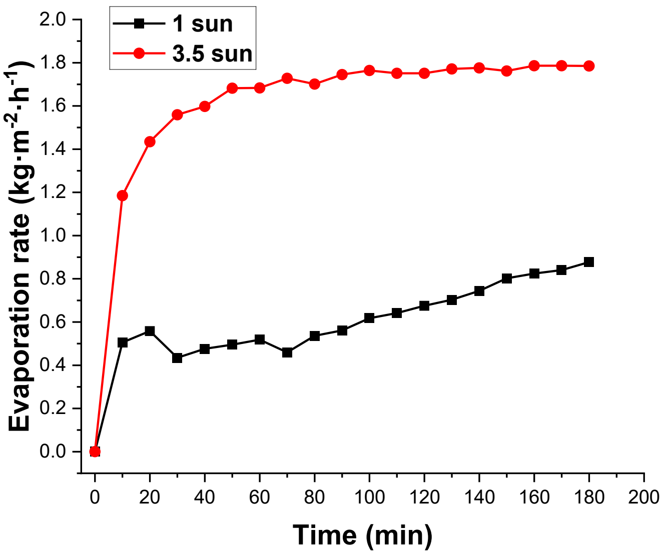

Figure S21. Time-dependent evaporation rates of the NP-CA nanofiber membrane in 10 wt% NaCl under continuous 3 h operation at 1 sun and 3.5 suns.

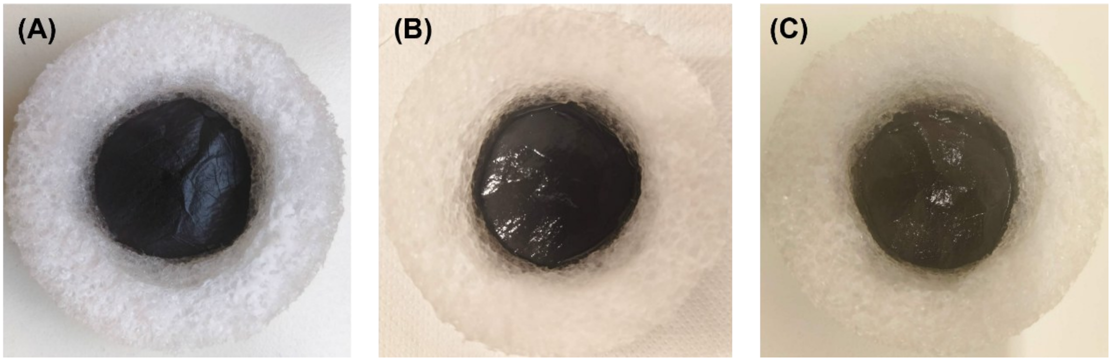

Figure S22. Photographs of the membrane before and after the continuous solar evaporation tests in 10 wt% NaCl: (A) before operation; (B) after 3 h at 1 sun; (C) after 3 h at 3.5 suns.

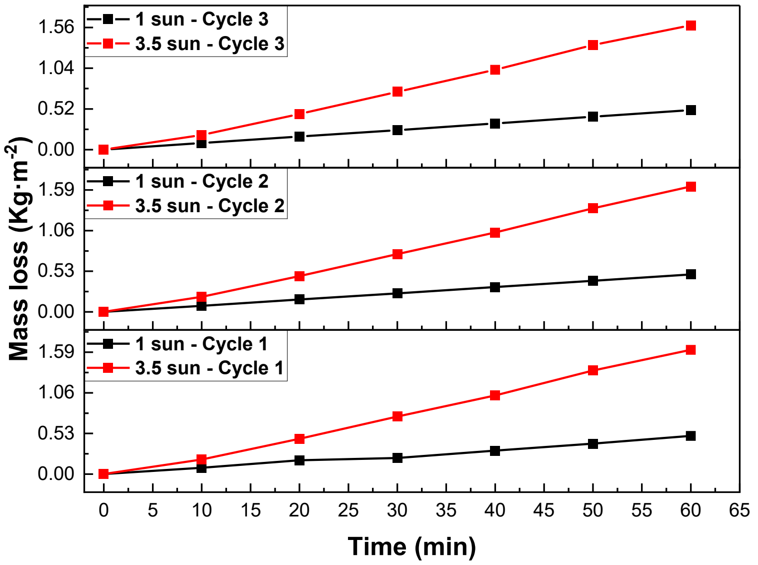

Figure S23. Repeated restart-based evaporation cycles of the NP-CA membrane in 10 wt% NaCl at 1 sun and 3.5 suns, showing mass loss for consecutive 1 h cycles.

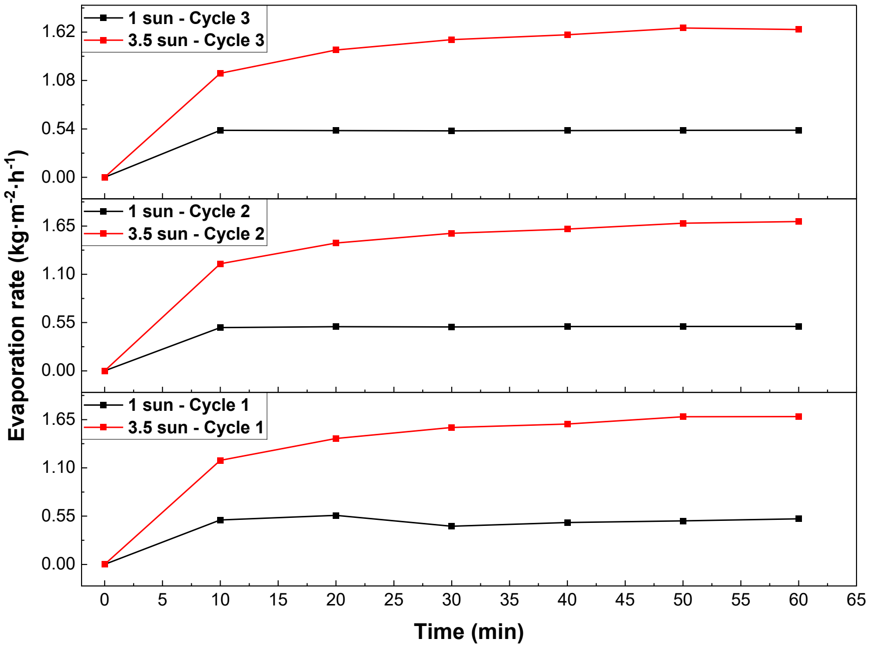

Figure S24. Repeated restart-based evaporation cycles of the NP-CA membrane in 10 wt% NaCl at 1 sun and 3.5 suns, showing time-dependent evaporation rates for consecutive 1 h cycles.

Table S2. Parameters and calculated solar-to-vapor efficiencies for the NP-coated evaporators under 1 sun and 3.5 suns. The solar-to-vapor efficiency η was calculated using Eq. 4 in the main text.

	**Cellulose blank**	**NP-nanofiber**	**Cellulose blank**	**NP-nanofiber**
**Evaporation rate under dark conditions**	**0.084 kg·m^{-2}·h^{-1}**			
**Net water evaporation rate m_{net}** (dark evaporation subtracted)	**0.354 kg·m^{-2}·h^{-1}**	**2.316 kg·m^{-2}·h^{-1}**	**0.076 kg·m^{-2}·h^{-1}**	**0.471 kg·m^{-2}·h^{-1}**
**Total phase change enthalpy h_{LV}**	**2.37×10^{6} J ·kg^{-1}**	**2.34×10^{6} J ·kg^{-1}**	**2.41×10^{6} J ·kg^{-1}**	**2.39×10^{6} J ·kg^{-1}**
**Solar optical concentration factor C_{opt}**	**3.5**	**3.5**	**1**	**1**
**Nominal direct solar irradiance q_{solar} under 1 sun**	**1 kW/m^2**	**1 kW/m^2**	**1 kW/m^2**	**1 kW/m^2**
**Solar-to-vapor efficiency η**	**6.66%**	**43%**	**5.08%**	**31.3%**

Table S3. The evaporation performance comparison between different literatures and this work.

Paper DOI	Cellulose substrate	Photothermal nanomaterial	NP loading amount (mg, total)	NP loading density (mg/cm^2)	1 sun evap. rate ($kg \cdot m^{-2} \cdot h^{-1}$)	1 sun efficiency (%)	Blank substrate rate ($kg \cdot m^{-2} \cdot h^{-1}$)	Blank efficiency (%)	Enhancement factor
10.1039/d5mh01166c	Cellulose paper (CP)	Iron oxide-graphene	7.1 wt% (mass fraction)	-	1.82	99	0.49	22.7	4.36
10.1002/advs.202205809	Cellulose Nanofibers (CNF)	Borophene	5	0.4	1.45	91.5	0.5	31.5	2.90
10.1002/agt2.531	MCE membrane	Hafnium Carbide	-	0.1	1.4	96	0.38	26.1	3.68
10.1016/j.jcis.2021.08.043	Cigarette Filter (CA)	MXene (Ti_3C_2Tx)	0.0417 g/g (mass fraction)	-	3.38	132.9	1	39.3	3.38
10.1002/adfm.202212301	Cellulose filter paper (Support)	TiN NPs / semi-rGO	-	0.0875 (TiN)	1.76	99.1	1.03	65	1.52
10.1021/acsomega.1c05348	Filter Paper (FP)	Candle Soot	-	-	1.16	75.1	0.55	35	2.15
10.3390/nano13061003	Cellulose filter paper	Au Nanoparticles	1	0.44	0.96	67	0.5	34.9	1.92
10.1002/smll.202505931	Cellulose paper	Co-doped Graphene	-	-	1.75	92.5	0.69	38.5	2.40
10.1016/j.jcis.2024.06.248	Cellulose Filament (CF)	MXene (Ti_3C_2Tx)	6.68	0.29	1.11	82.52	0.45	33	2.50
10.1016/j.solmat.2023.112597	Cellulose Acetate (d-CA)	Graphene	-	3 wt% (Conc.)	1.38	88.4	0.35	16.4	5.39
10.1039/d0ra07746a	Filter Paper (Whatman 42)	Activated Carbon (AC) + PEI	50	5.2	1.27	85.66	0.9	40.5	2.12
10.1016/j.cej.2023.143689	Cellulose fiber paper	Coal-based carbon nanomaterial	-	-	1.4	90.1	0.8	51.5	1.75
10.1016/j.jtice.2024.105398	Filter Paper (Advantec)	PbS dendrites / Graphene	45	-	1.39	86.8	0.69	43	2.02
10.1039/D2TA04365C	Commercial hydrophilic cellulose paper (22×8 cm sheet used for folding)	Polypyrrole	179	1.017	2.67	180	1.17	79	2.28
This work	Cellulose Acetate nanofiber	FeMnB nanofiber - 1sun	23.1	1.31	0.56	31.3	0.16	5.08	6.16
This work	Cellulose Acetate nanofiber	FeMnB nanofiber - 3.5 sun	23.1	1.31	2.4 (3.5 sun)	43.0 (3.5 sun)	0.438 (3.5 sun)	6.66 (3.5 sun)	6.46

Table S4. Fe, Mn and B concentration in condensed water samples from the 5 wt% and 10 wt% NaCl solutions, and of a pure milliQ water sample, measured with ICP-MS. All values ensure safety for potable use.

Sample	Fe (ppb)	Mn (ppb)	B (ppb)
milliQ water (untreated)	0.1	0.9	27
Condensed water from 5 wt% NaCl solution	0.3	4.3	291
Condensed water from 10 wt% NaCl solution	3.4	1.9	250
